# Peripheral tolerance by Treg via constraining OX40 signal in autoreactive T cells against desmoglein 3, a target antigen in pemphigus

**DOI:** 10.1073/pnas.2026763118

**Published:** 2021-11-30

**Authors:** Hisato Iriki, Hayato Takahashi, Naoko Wada, Hisashi Nomura, Miho Mukai, Aki Kamata, Hiromi Ito, Jun Yamagami, Takeshi Matsui, Yutaka Kurebayashi, Setsuko Mise-Omata, Hiroshi Nishimasu, Osamu Nureki, Akihiko Yoshimura, Shohei Hori, Masayuki Amagai

**Affiliations:** ^a^Department of Dermatology, Keio University School of Medicine, Tokyo 160-8582, Japan;; ^b^Laboratory for Skin Homeostasis, RIKEN Center for Integrative Medical Sciences, Yokohama City 230-0045, Japan;; ^c^Laboratory for Evolutionary Cell Biology of the Skin School of Bioscience and Biotechnology, Tokyo University of Technology, Tokyo 192-0982, Japan;; ^d^Department of Pathology, Keio University School of Medicine, Tokyo 160-8582, Japan;; ^e^Department of Microbiology and Immunology, Keio University School of Medicine, Tokyo 160-8582, Japan;; ^f^Department of Biological Science, Graduate School of Science, The University of Tokyo, Tokyo 113-0033, Japan;; ^g^Structural Biology Division, Research Center for Advanced Science and Technology, The University of Tokyo, Tokyo 153-8904, Japan;; ^h^Graduate School of Pharmaceutical Sciences, The University of Tokyo, Tokyo 113-0033, Japan

**Keywords:** peripheral immunological tolerance, regulatory T cells, Foxp3, OX40, autoreactive T cells

## Abstract

Antigen-specific peripheral tolerance is crucial to prevent the development of organ-specific autoimmunity. However, its function decoupled from thymic tolerance remains unclear. We used desmoglein 3 (Dsg3), a pemphigus antigen expressed in keratinocytes, to analyze peripheral tolerance under physiological antigen-expression conditions. Dsg3-deficient thymi were transplanted into athymic mice to create a unique condition in which Dsg3 was expressed only in peripheral tissue but not in the thymus. When bone marrow transfer was conducted from high-avidity Dsg3-specific T cell receptor–transgenic mice to thymus-transplanted mice, Dsg3-specific CD4^+^ T cells developed in the transplanted thymus but subsequently disappeared in the periphery. Additionally, when Dsg3-specific T cells developed in *Dsg3*^−/−^ mice were adoptively transferred into Dsg3-sufficient recipients, the T cells disappeared in an antigen-specific manner without inducing autoimmune dermatitis. However, Dsg3-specific T cells overcame this disappearance and thus induced autoimmune dermatitis in Treg-ablated recipients but not in *Foxp3*-mutant recipients with dysfunctional Tregs. The molecules involved in disappearance were sought by screening the transcriptomes of wild-type and *Foxp3*-mutant Tregs. *OX40* of Tregs was suggested to be responsible. Consistently, when *OX40* expression of Tregs was constrained, Dsg3-specific T cells did not disappear. Furthermore, Tregs obtained OX40L from dendritic cells in an *OX40*-dependent manner in vitro and then suppressed OX40L expression in dendritic cells and Birc5 expression in Dsg3-specific T cells in vivo. Lastly, CRISPR/Cas9-mediated knockout of *OX40* signaling in Dsg3-specific T cells restored their disappearance in Treg-ablated recipients. Thus, Treg-mediated peripheral deletion of autoreactive T cells operates as an *OX40*-dependent regulatory mechanism to avoid undesired autoimmunity besides thymic tolerance.

## Significance

Immune tolerance is crucial to prevent harmful immune reactions against self-antigens and well operated by central thymic tolerance and peripheral tissue tolerance. However, peripheral tolerance had been investigated under influence from thymic tolerance. We successfully decoupled peripheral tolerance from thymic tolerance by utilizing autoantigen-deficient thymus. Experiments revealed that self-antigen presentation in steady state initiated proliferation but subsequent disappearance of autoreactive CD4^+^ T cells in draining lymph nodes. After screening of representative candidates, including Ctla4, autoimmune regulator, and Pd-1, the mechanism was found to depend on regulatory T cell (Treg) function that constrained OX40 signaling of the T cells. This study presented fundamental, but potent, Treg-mediated tolerance mechanisms of peripheral tissues to prevent autoimmunity as compensatory roles for central tolerance.

Immunological tolerance is crucial for maintaining immune homeostasis and preventing autoimmune diseases in healthy individuals. Therefore, identifying the mechanism of tolerance in normal subjects is essential to understanding disease pathology and has the potential to lead to novel approaches to treating intractable autoimmune diseases. Classic studies have demonstrated that most thymocytes undergo deletion or anergy after encountering autoantigens in the thymus (1, 2). Such thymus-mediated central tolerance is not necessarily perfect (3). In addition, the human thymus begins to decrease in size and activity by the early teens and is gradually replaced by adipose tissue; it loses its functionality by 50 y of age (4). Therefore, it may be important that the immune system should maintain another tolerance mechanism in peripheral tissue to constrain autoimmunity; this is known as peripheral tolerance.

Roles of peripheral immune tolerance have previously been investigated in various experimental conditions, and it was reported that several immunoregulatory molecules, such as PD-1, CTLA-4, and IL-10, play pivotal roles in the periphery (5–7). However, many of these experiments were conducted without completely excluding the influence of the thymus. For precise analysis of peripheral tolerance, both central and peripheral tolerance mechanisms that engage in mutual compensation to avoid harmful autoimmunity must be considered. In the thymus, medullary thymic epithelial cells (TECs) promiscuously express peripheral tissue-specific antigens via transcription factors such as autoimmune regulator (Aire) and forebrain embryonic zinc finger 2 (Fezf2) (8–10). Dual antigen expression in the thymus and peripheral tissue would hamper appropriate interpretation as to which tolerance contributes to experimental results, central or peripheral tolerance.

Previous experiments designed to elucidate central tolerance have also faced the same issue. A tissue-specific promoter such as the keratin 4 promoter was used to express the antigen in TECs to ensure that T cells encounter the autoantigen in the thymus (11, 12). However, the antigen was also simultaneously expressed in peripheral tissue, and central tolerance was evaluated without considering the contribution of peripheral tolerance. Thus, a complete picture of peripheral tolerance mechanisms remains unclarified, and its understanding is practically more helpful in terms of future clinical application, since molecules and cells critically working in peripheral tolerance is more accessible than those of central tolerance. Therefore, it is necessary to prepare an experimental design that decouples peripheral tolerance from central tolerance.

Dsg3 is an adhesion molecule expressed in keratinocytes and a target autoantigen of pemphigus vulgaris (PV), an autoimmune bullous disease (13). A series of studies demonstrated that Dsg3-specific T cell receptor–transgenic T cells (Dsg3H1 T cells) directly infiltrated the epidermis and induced cellular immunity to Dsg3-bearing keratinocytes after adoptive transfer into *Rag2^−/−^* mice, leading to skin inflammation known as interface dermatitis (14). Interface dermatitis is the pathological changes that are observed in paraneoplastic pemphigus, one of the subtypes of pemphigus with anti-Dsg3 autoimmunity (15) as well as lupus erythematosus, lichen planus, and graft versus host disease (16). We used interface dermatitis as a disease model to analyze how antigen-specific autoimmunity is constrained by peripheral tolerance in this study.

Our goal was to define the mechanism of peripheral tolerance to desmoglein 3 (Dsg3), an epidermal autoantigen of PV. To eliminate contributions of central tolerance, we transplanted the thymus from Dsg3-deficient mice to athymic nude mice. This model allows us to evaluate and dissect the peripheral tolerance in a way previous classic studies could not. We found that key contributors that maintain peripheral tolerance are regulatory T cells (Tregs). This mechanism operates in a Dsg3-specific manner, and its failure results in the induction of autoimmune status. These findings provide a framework for understanding the mechanisms of peripheral tolerance and developing a therapeutic strategy to eliminate autoreactive T cells by modifying Tregs as antigen-specific immune suppression.

## Results

### Dsg3-Specific TCR Tg T Cells Are Deleted in the Dsg3-Bearing Thymus.

**Fig. 1.**
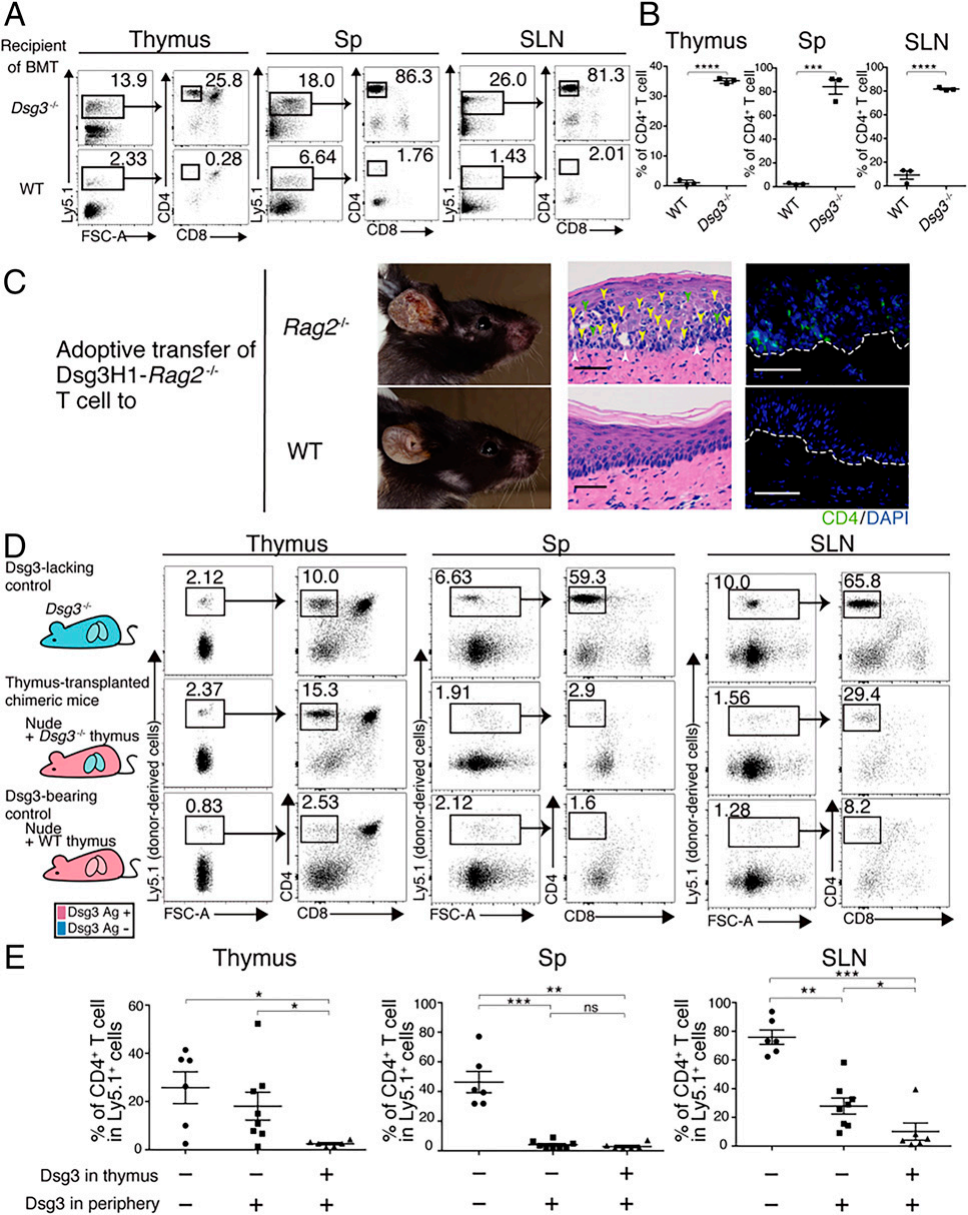
Thymus transplantation model of Dsg3-dependent peripheral immune regulation. (*A* and *B*) Flow cytometry (FCM) plots and quantitative summaries of thymocytes and lymphocytes in the Sp and SLNs showing the deletion of Dsg3H1-*Rag2*^−/−^ T cells in the Dsg3-expressing thymus (gated on Ly5.1^+^). The proportion of Ly5.1^+^ cells in each organ are also shown. Data are from two independent experiments (*n* = 3 mice per group). (*C*) Clinical phenotype and pathology of the palate of *Rag2*^−/−^ and WT mice administered Dsg3H1-*Rag2*^−/−^ T cells. In the H&E-stained images, infiltrating lymphocytes (yellow arrow), Civatte body (green), and liquefaction (white) are indicated. For immunofluorescence (IF), the palate was stained with anti-CD4 Abs (green) and DAPI (blue). Dotted lines indicate the basement membrane zone (BMZ). (Scale bars, 50 μm.) Data are from three independent experiments (*n* = 3 mice per group). (*D*) The Dsg3 expression pattern of recipient mice. Presence (pale red) and absence (blue) of Dsg3, and FCM plots of thymocytes and lymphocytes in recipient mice 2 mo after BMT. FCM plots indicating the proportions of Ly5.1^+^ cells are also shown (left column of each tissue). FCM plots showing CD4 versus CD8 T cell development are shown after gating on Ly5.1^+^ cells (right column of each tissue). The experimental approach is described in *SI Appendix*, Fig. S1*C*. Data are from three to five experiments independent experiments (*n* = 1 to 3 mice per group). (*E*) Proportion of Dsg3H1-*Rag2*^−/−^ T cells in the thymus, SLNs, and Sp of each recipient mouse (*n* = 6 or 8; the data of three experiments were pooled). Means ± SEMs are shown. *P < 0.05, **P < 0.01, and ***P < 0.001 based on unpaired *t* tests (*B*) or a Mann–Whitney *U* test (*E*) between the groups.

To investigate the mechanisms of peripheral tolerance to Dsg3, we used Dsg3-specific T cell receptor (TCR) Tg mice, hereafter “Dsg3H1 mice” (14). In this study, Dsg3H1 mice were crossed with *Rag2*^−/−^ mice to exclude endogenous TCR expression and ensure that CD4^+^ T cells expressed only Dsg3-specific TCR. Bone marrow cells of Dsg3H1-*Rag2*^−/−^ mice were transplanted into wild-type (WT) and *Dsg3*^−/−^ mice after irradiation with 7.5 Gy; the fate of Dsg3H1-*Rag2*^−/−^ T cells was then observed. Two months after bone marrow transplantation (BMT), Dsg3H1-*Rag2*^−/−^ T cells were evident as CD4 single positive (CD4SP) cells in the thymi of *Dsg3*^−/−^ mice (Fig. 1 *A* and *B*) and in peripheral lymphoid organs. In contrast, neither CD4SP cells nor mature CD4^+^ T cells were detected in the thymus or periphery of Dsg3-bearing WT mice, respectively (Fig. 1 *A* and *B*). This indicated that Dsg3H1-*Rag2*^−/−^ T cells were deleted via negative selection in the WT thymus. To evaluate pathogenicity of Dsg3H1-*Rag2*^−/−^ CD4^+^ T cells, Dsg3H1-*Rag2*^−/−^ CD4^+^ T cells that had developed without undergoing Dsg3-specific negative selection in *Dsg3*^−/−^ mice were isolated from peripheral lymph nodes (LNs) or spleen (Sp) and transferred to *Rag2*^−/−^ immunodeficient mice; subsequently, Dsg3H1-*Rag2*^−/−^ CD4^+^ T cells proliferated under lymphopenic conditions, and the recipients developed skin inflammation pathologically classified as interface dermatitis (Fig. 1*C*). However, when the Dsg3H1-*Rag2*^−/−^ T cells were adoptively transferred into WT mice, disease development was completely inhibited (Fig. 1*C*). Therefore, Dsg3H1-*Rag2*^−/−^ T cells represent pathogenic autoimmune CD4^+^ T cells, but their pathogenicity is efficiently constrained by physiological mechanisms in WT mice that we further investigated in this study.

### Dsg3-Dependent Immune Regulation by Peripheral Organs Is Responsible for the Disappearance of Autoreactive CD4^+^ T Cells.

We created a model to study peripheral tolerance to Dsg3 without influence of central tolerance. The Dsg3-deficient thymus from *Dsg3*^−/−^ mice was transplanted to athymic nude mice to generate a unique chimeric condition in terms of Dsg3 expression (Fig. 1 *D*, middle row and *SI Appendix*, Fig. S1*A*), in which Dsg3 was deficient in the thymus but was expressed in peripheral tissues, including the skin. In these mice, T cells were not subjected to Dsg3-specific central tolerance. The cells that developed in the Dsg3-deficient thymus are able to move into the secondary lymphoid organs and Dsg3-expressing peripheral tissues such as skin. In addition, we prepared two sets of controls: Dsg3-lacking and Dsg3-bearing controls (Fig. 1 *D*, top and bottom row). *Dsg3*^−/−^ mice were used for the Dsg3-lacking control; in these mice, Dsg3 was deficient in both the thymus and peripheral tissues. WT thymus-transplanted nude mice were used for the Dsg3-bearing control; in these mice, Dsg3 was expressed in both the thymus and peripheral tissues (*SI Appendix*, Fig. S1*A*). Transplanting the thymus to nude mice restored T cell development, as reported previously (17–19) (*SI Appendix*, Fig. S1*B*). Furthermore, to study Dsg3-specific CD4^+^ T cell development under the three conditions, we transferred bone marrow from Ly-5.1^+^ Dsg3H1-*Rag2*^−/−^ mice into the three kinds of recipients (*SI Appendix*, Fig. S1*C*). Two months later, the thymus, skin-draining LNs (SLNs), and Sp of each recipient were analyzed. Under BMT conditions optimized for the three recipients, small populations of Ly5.1^+^ donor-derived thymocytes were detected among CD4^−^CD8^−^ thymocytes at the developmental stage prior to negative selection, thus 7.80 ± 3.24% in Dsg3-lacking controls, 2.26 ± 0.86% in thymus-transplanted chimeric mice, and 2.25 ± 0.76% in Dsg3-bearing controls (*SI Appendix*, Fig. S1*D*). In recipient *Dsg3^−/−^* mice, Dsg3H1-*Rag2*^−/−^ CD4^+^ T cells developed in the thymus and became the major donor-derived population in the SLNs and Sp (Fig. 1 *D*, top row and *E*). In contrast, few Dsg3H1-*Rag2*^−/−^ CD4^+^ T cells were detected in the transplanted Dsg3^+/+^ thymus, and their proportions were negligible and significantly decreased in the Sp and SLNs, respectively, in Dsg3^+/+^ thymus-transplanted nude mice compared to *Dsg3^−/−^* controls (Fig. 1 *D*, bottom row and *E*). In terms of recipient-derived T cells, WT T cells developed into a CD4SP population in transplanted WT thymi of nude mice after sublethal irradiation prior to BMT (*SI Appendix*, Fig. S1*E*). These results suggest that the transplanted thymus maintains not only normal CD4^+^ T cell development but also the mechanism that deletes Dsg3-specific CD4^+^ T cells.

To investigate autoreactive T cell behavior upon first contact with cognate antigen in the peripheral lymphoid organ, we analyzed *Dsg3*^−/−^ thymus-transplanted nude mice after the transfer of bone marrow from Dsg3H1-*Rag2*^−/−^ mice. Dsg3H1-*Rag2*^−/−^ T cells developed abundantly to the CD4SP population in the thymus, as in *Dsg3*^−/−^ mice (Fig. 1 *D*, middle row and* E*). However, Dsg3H1-*Rag2*^−/−^ T cell populations were negligible or significantly decreased in the Sp and SLNs, respectively (Fig. 1 *D*, middle row and *E*). These results indicate that a peripheral regulatory mechanism eliminated Dsg3H1-*Rag2*^−/−^ T cells.

### The Adoptive Transfer Model Reproduces the Peripheral Disappearance of Dsg3H1-*Rag2*^−/−^ T Cells.

**Fig. 2.**
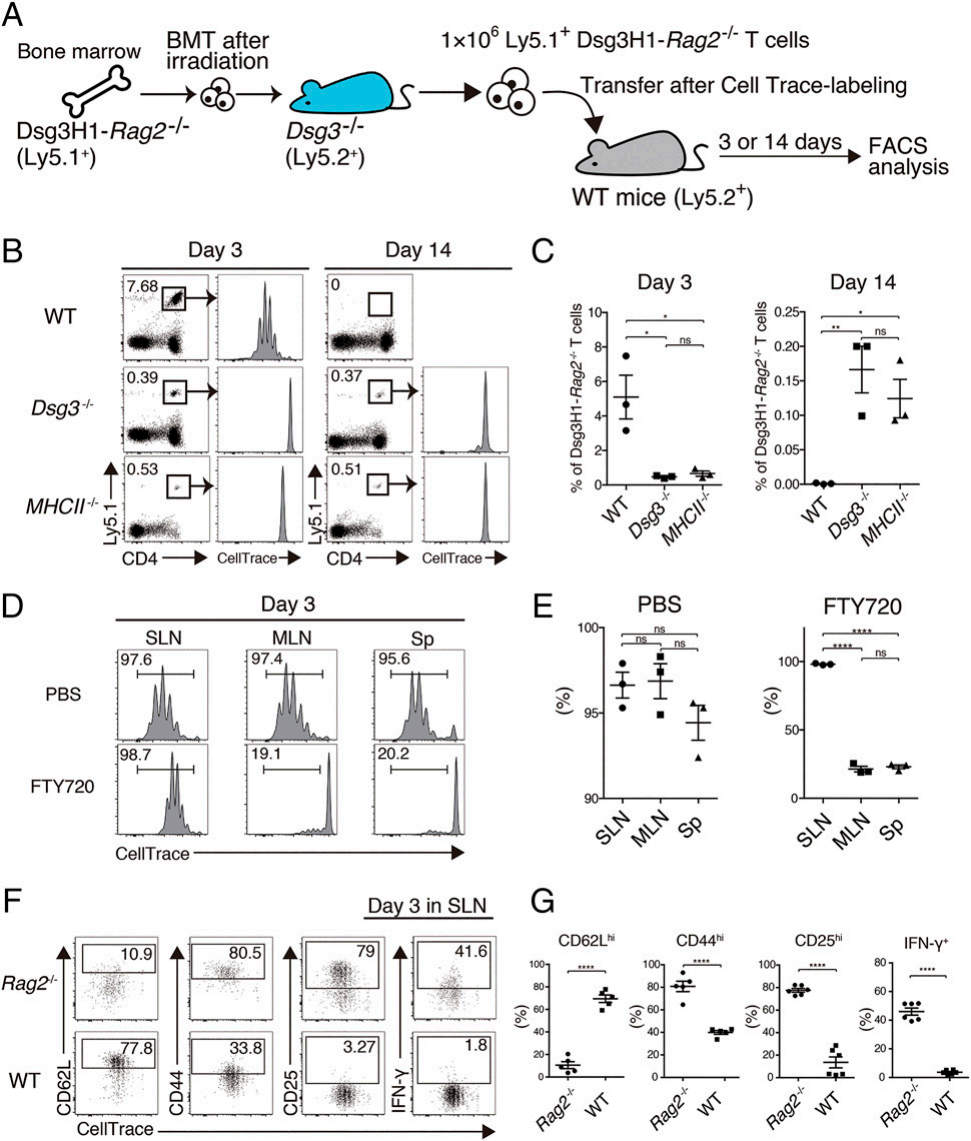
Adoptive transfer model showing the peripheral disappearance of Dsg3H1-*Rag2*^−/−^ T cells. (*A*) Outline of the adoptive transfer of Dsg3H1-*Rag2*^−/−^ T cells to WT mice. (*B* and *C*) FCM plots and quantitative summaries of lymphocytes in SLNs of WT, *Dsg3*^−/−^, and *MHCII*^−/−^ mice at days 3 and 14 after transfer (gated on Vβ6^+^). Dsg3H1-*Rag2*^−/−^ T cells were gated by square. (*D* and *E*) Histogram and quantitative summaries of intensity of cell proliferation tracer in proliferating Dsg3H1-*Rag2*^−/−^ T cells in the SLNs, MLNs, and Sp of the FTY720-injection and control groups 3 d after transfer. (*F* and *G*) FCM plots and quantitative summaries of the expression of the indicated factors in Dsg3H1-*Rag2*^−/−^ T cells in SLNs at day 3 after transfer to *Rag2*^−/−^ and WT mice (gated on Vβ6^+^Ly5.1^+^CD4^+^ cells). Means ± SEMs are shown. ns, not significant, *P < 0.05, **P < 0.01, and ****P < 0.0001 based on unpaired *t* tests between the groups. Data are from three (*B, C, F*, and *G*) or two (*D* and *E*) independent experiments (*n* = 3 to 5 mice per group).

To further investigate key subsets of immune cells and molecules responsible for the peripheral tolerance observed in the thymus transplantation model, we used the adoptive transfer method. Bone marrow was transferred from Dsg3H1-*Rag2*^−/−^ mice to *Dsg3*^−/−^ mice so that Dsg3H1-*Rag2*^−/−^ T cells developed in the thymus without exposure to Dsg3. Next peripheral Dsg3H1-*Rag2*^−/−^ T cells were isolated from the Sp and LNs of recipient *Dsg3*^−/−^ mice and adoptively transferred into WT mice (Fig. 2*A*). By using mature Dsg3H1-*Rag2*^−/−^ T cells from the Sp and LNs, we were able to focus on peripheral immune regulation without considering thymic regulation in the recipient mice.

In this approach, Dsg3-specific T cells first encounter Dsg3 in the periphery, as in the thymus transplantation model. At day 14 after transfer, Dsg3H1-*Rag2*^−/−^ T cells were not detected in SLNs, the Sp, MLNs, or Dsg3-bearing peripheral tissues such as skin and were therefore considered to have disappeared in recipient WT mice (Fig. 2 *B*, *Top* and *C* and *SI Appendix*, Fig. S2*A*); whereas Dsg3H1-*Rag2*^−/−^ T cells remained in the recipient *Dsg3*^−/−^ mice (Fig. 2 *B*, middle row and *C*). However, when Ly5.1 WT CD4^+^ T cells were transferred to WT mice, the transferred cells remained (*SI Appendix*, Fig. S2*B*). These results indicate that the adoptive transfer model at least partially mimics the thymus transplantation model and enables investigation of the mechanisms of Dsg3-specific peripheral tolerance using recipient mice under various experimental conditions.

### Presentation of Dsg3 Is Necessary for the Peripheral Disappearance of Dsg3H1-*Rag2*^**−/−**^ T Cells in SLNs.

Next, we determined whether the presentation of Dsg3 is involved in peripheral deletion; for this purpose, we used *MHC class II*^−/−^ (*MHCII*^−/−^) mice as recipients (Fig. 2 *B*, bottom row and *C*). After adoptive transfer, Dsg3H1-*Rag2*^−/−^ T cells failed to disappear at day 14, which indicates that MHCII-dependent antigen presentation is necessary for the peripheral disappearance of Dsg3H1-*Rag2*^−/−^ T cells.

Next Dsg3H1-*Rag2*^−/−^ T cells were labeled with cell proliferation tracer and observed 3 d after transfer. Dsg3H1-*Rag2*^−/−^ T cells proliferated vigorously in WT mice but negligibly in *Dsg3^−/−^* and *MHCII^−/−^* mice (Fig. 2 *B* and *C* and *SI Appendix*, Table S1). Furthermore, FTY720 treatment on WT mice, which trapped lymphocytes in the secondary lymphoid tissue, enabled Dsg3H1-*Rag2*^−/−^ T cells to proliferate in the SLNs but to a lesser degree in the Sp and MLNs (Fig. 2 *D* and *E*). These results indicate that Dsg3H1-*Rag2*^−/−^ T cells proliferate before disappearing upon antigen presentation in the SLNs of WT mice.

### Dsg3H1-*Rag2*^**−/−**^ T Cells Show Aberrant Activation during Antigen-Specific Peripheral Disappearance.

T cells are typically activated after antigen recognition. However, most Dsg3H1-*Rag2*^−/−^ T cells maintained a so-called naïve phenotype (CD62L^hi^ and CD44^lo^) and did not up-regulate the activation marker, CD25, during proliferation after transfer to WT mice (Fig. 2 *F* and *G*). By contrast, Dsg3H1-*Rag2*^−/−^ T cells acquired a memory phenotype (CD62L^lo^ and CD44^hi^) and up-regulated CD25 during homeostatic proliferation after transfer to *Rag2*^−/−^ mice, in which the autoreactive T cells became pathogenic and induced interface dermatitis (Fig. 2 *F* and *G*). IFN-γ is a key cytokine for Dsg3H1 T cells to induce interface dermatitis (14), but its expression in Dsg3H1-*Rag2*^−/−^ T cells was significantly decreased after transfer to WT mice (Fig. 2 *F* and *G*), which indicates that Dsg3H1-*Rag2*^−/−^ T cells are less functional as effector T cells during the disappearance. Thus, Dsg3H1-*Rag2*^−/−^ T cells proliferate after antigen recognition but failed to acquire an activated phenotype, resulting in disappearance under physiological conditions in the periphery of WT mice. These results imply that other steps aside from antigen recognition that are necessary for full activation of T cells are missing during the disappearance. Costimulatory signals are one of the representative steps for proper T cell activation.

### Foxp3^+^ Regulatory T Cells Are Indispensable for the Peripheral Disappearance of Dsg3H1-*Rag2*^−/−^ T Cells and Prevention of Skin Inflammation.

Next, we sought to identify molecules responsible for the peripheral disappearance of Dsg3H1-*Rag2*^−/−^ T cells. Of the several molecules associated with tolerance, Aire and PD-1 are representative; extrathymic Aire-expressing cells and PD-1 signaling are reportedly essential for antigen-specific CD8^+^ T cell deletion in pancreatic LNs and the liver, respectively (20, 21). However, Dsg3H1-*Rag2*^−/−^ T cells disappeared after adoptive transfer into *Aire*^−/−^ mice and anti-PD-1–treated WT mice, which suggests no involvement of Aire and PD-1 in the disappearance (*SI Appendix*, Fig. S3 *A* and *B*). Next, to perform unbiased screening for responsible molecules, we planned to identify the cells responsible for the disappearance and then utilize their transcriptome data.

**Fig. 3.**
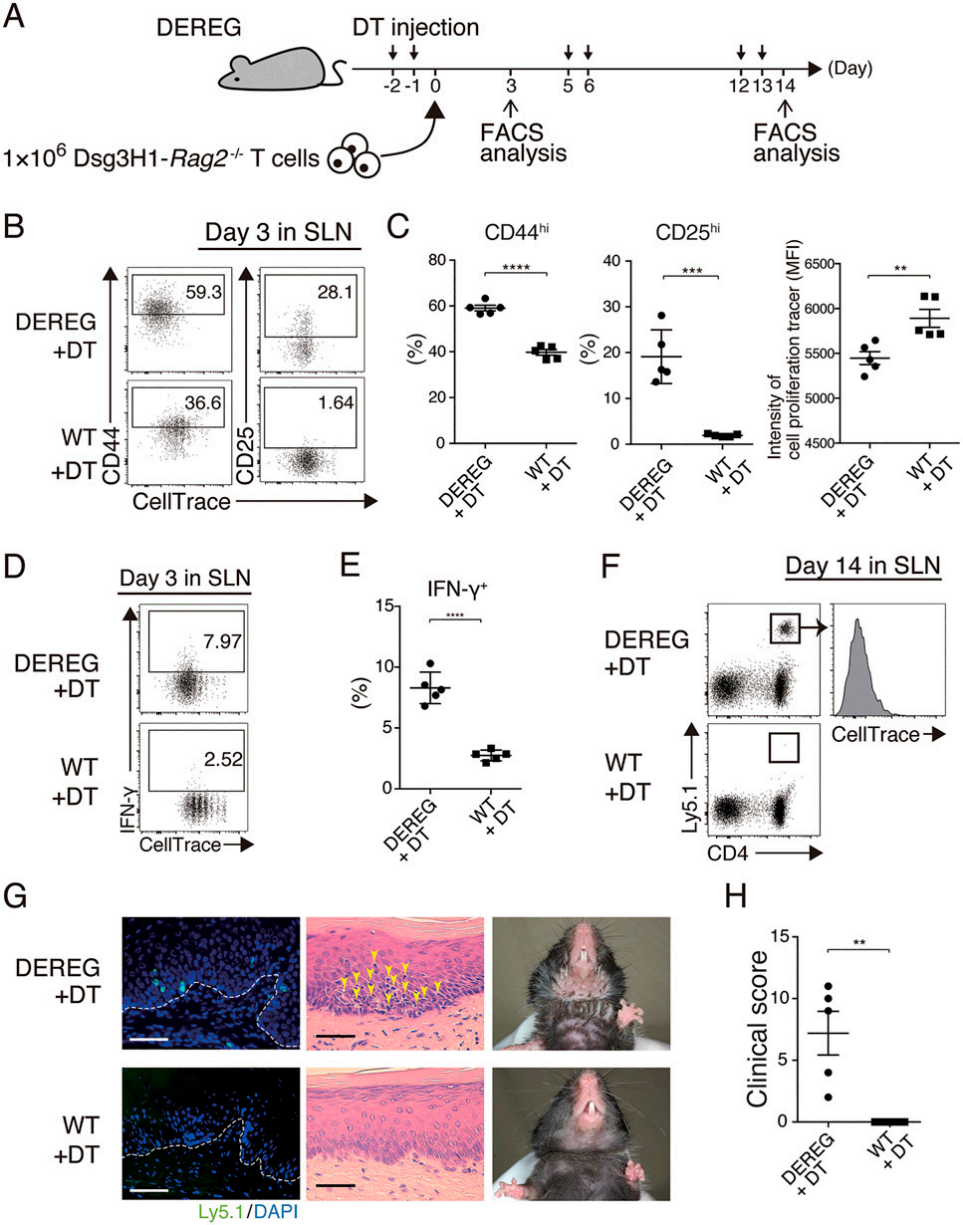
Dsg3H1-*Rag2*^−/−^ T cells are activated and induce interface dermatitis when Tregs are ablated. (*A*) Outline of the adoptive transfer of Dsg3H1-*Rag2*^−/−^ T cells when Treg is ablated. (*B–E*) FCM plots and quantitative summaries of the expression and the intensity of the indicated factors in Dsg3H1-*Rag2*^−/−^ T cells in SLNs at day 3 after transfer to DT-treated DEREG and control mice (gated on Vβ6^+^Ly5.1^+^CD4^+^). (*F*) FCM plots of lymphocytes in SLNs of WT and DT-treated DEREG mice at day 14 after transfer (gated on Vβ6^+^). Ly5.1^+^CD4^+^ cells are Dsg3H1-*Rag2*^−/−^ T cells. (*G*) Clinical phenotype and pathology of the palate of DT-treated DEREG and WT mice at day 14 after the transfer of Dsg3H1-*Rag2*^−/−^ T cells. For IF, the palate was stained with anti-Ly5.1 Ab (green) and DAPI (blue). Dotted lines indicate the BMZ. Yellow arrow indicates infiltrating lymphocytes. (Scale bars, 50 μm.) (*H*) Clinical scores of DT-treated DEREG and WT mice at day 14 after the transfer of Dsg3H1-*Rag2*^−/−^ T cells. Means ± SEMs are shown. **P < 0.01, ***P < 0.001, and ****P < 0.0001 by unpaired *t* tests between the groups. Data are from at least three independent experiments (*n* = 3 to 5 mice per group).

First, we investigated the potential role of Foxp3^+^ Tregs, which are essential for maintaining immune homeostasis. To evaluate the importance of Tregs in the peripheral disappearance of Dsg3H1-*Rag2*^−/−^ T cells, we used DEREG mice, in which diphtheria toxin (DT) receptor is expressed in Foxp3^+^ T cells, which can be transiently deleted by DT injection (22). When Dsg3H1-*Rag2*^−/−^ T cells were transferred to Treg-ablated DEREG mice (Fig. 3*A*), proliferation of Dsg3H1-*Rag2*^−/−^ T cells was observed in the SLNs at day 3 (Fig. 3*B*). Moreover, Dsg3H1-*Rag2*^−/−^ T cells in Treg-ablated mice exhibited greater proliferative response and up-regulation of CD25 and CD44 expression than that in WT mice and showed an typical activation phenotype (Fig. 3 *B* and *C*). In addition, Dsg3H1-*Rag2*^−/−^ T cells produced more IFN-γ in Treg-ablated mice than that in WT mice (Fig. 3 *D* and *E*). These results indicate that Tregs constrain normal activation during the disappearance of Dsg3H1-*Rag2*^−/−^ T cells.

Indeed, Dsg3H1-*Rag2*^−/−^ T cells not only increased but remained a significant population after proliferation at day 14 in Treg-ablated mice, while Dsg3H1-*Rag2*^−/−^ T cells disappeared in DT-treated WT mice as control (Fig. 3*F*). Furthermore, autoreactive T cells induced the pathogenic phenotype by infiltrating the palate and skin (Fig. 3*G*) with higher clinical score (*SI Appendix*, Table S2) than that of DT-treated WT mice (Fig. 3*H* and *SI Appendix*, Table S1), while the interface dermatitis was not clearly observed in mild skin inflammation endogenously occurred just after Treg depletion (*SI Appendix*, Fig. S4 *A* and *B*). These results indicate that Tregs were required for Dsg3-specific CD4^+^ T cell disappearance in the periphery.

### Deletion of Dsg3H1-*Rag2*^**−/−**^ T Cells Is Maintained in the Periphery of Immunodysregulation, Polyendocrinopathy Enteropathy X-Linked– Derived *Foxp3* Mutant Mice.

Given that Tregs are crucial for maintaining Dsg3-specific peripheral tolerance, we next attempted to identify Treg-derived factors involved in this process and extract one of the responsible pathways affected in disappearing autoreactive T cells. To this end, we focused on patients with immunodysregulation, polyendocrinopathy enteropathy X-linked syndrome, a rare disease linked to dysfunction in Foxp3 (23). Three mutations in *Foxp3* (A384T, I363V, and R397W) are associated with disease severity. In these three *Foxp3* mutation-knock in mice, Foxp3^+^ Treg function was affected to various degrees (24). Therefore, these three *Foxp3* mutant strains were used as recipients, and the fate of Dsg3H1-*Rag2*^−/−^ T cells in the periphery was evaluated in each strain after adoptive transfer.

**Fig. 4.**
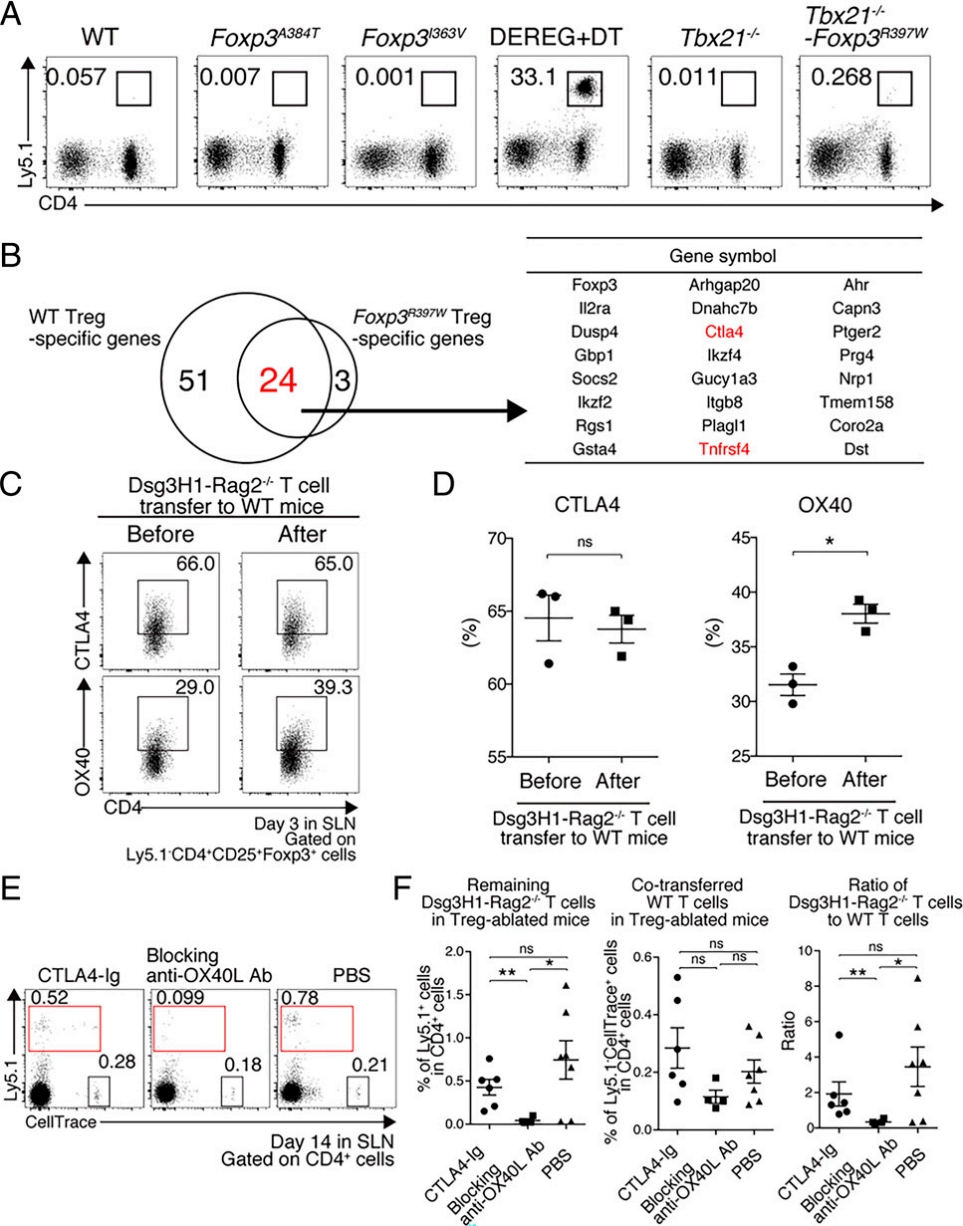
OX40 on Tregs is required for the peripheral disappearance of Dsg3H1-*Rag2*^−/−^ T cells. (*A*) FCM plots of lymphocytes in SLNs of WT, *Foxp3^A384T^*, *Foxp3^I363V^*, *Tbx21*^−/−^, *Tbx21^−/−^Foxp3^I363V^*, and DT-treated DEREG mice at day 14 after transfer (gated on Vβ6^+^ cells). Dsg3H1-*Rag2*^−/−^ T cells were gated by squares. Data are from three independent experiments (*n* = 1 to 3 mice per group). (*B*) Venn diagrams showing WT Treg-specific genes (left circle) and *Foxp3^R397W^* Treg-specific genes (right circle). The expression of 24 genes was more than fivefold higher in WT and *Foxp3^R397W^* Tregs than in Tconvs. (*C* and *D*) FCM plots and quantitative summaries of CTLA-4 and OX40 expression in Tregs from SLNs before and 3 d after the transfer of Ly5.1^+^ Dsg3H1-*Rag2*^−/−^ T cells (gated on Ly5.1^−^CD4^+^CD25^+^Foxp3^+^). Data are from three independent experiments (*n* = 3 to 6 mice per group). (*E* and *F*) FCM plots and quantitative summaries of lymphocytes in SLNs of CTLA4-Ig, anti-OX40L Ab, and PBS-injected DT-treated DEREG mice at day 14 after the transfer of 1 × 10^6^ CellTrace-labeled Ly5.1^+^ Dsg3H1-*Rag2*^−/−^ T ells and 1 × 10^6^ Ly5.2^+^ WT T cells as an internal control (gated on CD4^+^). Proliferated CellTrace^low^Ly5.1^+^Dsg3H1-*Rag2*^−/−^ T cells and CellTrace^+^Ly5.1^−^ cotransferred WT T cells were gated by red and black squares. Remaining Dsg3H1-*Rag2*^−/−^ T cell ratio was calculated as the proportion of CellTrace^low^Ly5.1^+^Dsg3H1-*Rag2*^−/−^ T cells divided by that of CellTrace^+^Ly5.1^−^ WT T cells. The data of three independent experiments were pooled (*n* = 1 to 3). Means ± SEMs are shown. ns, not significant, *P < 0.05 and **P < 0.01 based on unpaired unpaired *t* tests (*D*) or a Mann–Whitney *U* test (*F*) between the groups.

*Foxp3^I363V/Y^* and *Foxp3^A384T/Y^* mice showed signs of mild-to-moderate tissue inflammation, including dermatitis, by 3 mo of age. When transferred into 4-wk-old mice, Dsg3H1-*Rag2*^−/−^ T cells disappeared at day 14 in both strains (Fig. 4*A*). *Foxp3^R397W/Y^* mice, which showed the most severe phenotype among the three strains, died at around 6 wk because of aberrant systemic inflammation, similar to *Foxp3*-deficient *scurfy* mice (25). Therefore, we crossed *Foxp3^R397W/Y^* mice with *Tbx21*^−/−^ mice to prolong survival (26) and used *Foxp3^R397W/Y^Tbx21*^−/−^ mice as recipients at the age of 6 wk. In this mutant, most Dsg3H1-*Rag2*^−/−^ T cells disappeared on day 14 (Fig. 4*A*), even though *Foxp3^R397W/Y^Tbx21*^−/−^ mice had already developed dermatitis before the transfer (*SI Appendix*, Fig. S4*C*). Therefore, the *Foxp3^R397W^* mutation severely affected Treg function in a manner similar to *scurfy* Tregs but did not significantly impact the peripheral disappearance of Dsg3H1-*Rag2*^−/−^ T cells. That is, the downstream target molecules regulated by Foxp3 were not directly linked to the peripheral disappearance of Dsg3H1-*Rag2*^−/−^ T cells, although Foxp3^+^ Tregs themselves are required for the deletion, as seen in DT-treated DEREG mice. This led us to hypothesize that peripheral deletion may be linked to Foxp3-independent molecular events that operate in Foxp3^+^ Tregs.

### CTLA-4 and OX40 Are Up-Regulated in Tregs in a Mutant Foxp3-Independent Manner.

Because the deletion of Dsg3H1-*Rag2*^−/−^ T cells occurred in the three *Foxp3* mutant strains but not in the Treg-ablated mice, Treg-specific molecules functional in all of the mutants could have been responsible for the peripheral tolerance to Dsg3. Therefore, we used the global gene expression database (GEO; GSE89744) to identify candidate factors among those obtained from the mutant and WT Tregs (24). As the gene expression profiles of *Foxp3^R397W^* and *Foxp3^I363V^* Tregs are reportedly similar to that of conventional T cells (Tconv), but very different from that of WT Tregs (24), the numbers of genes commonly up-regulated in both WT and *Foxp3^R397W^* Tregs (compared to Tconv) were limited; only 24 genes were candidates for deletional tolerance (Fig. 4*B*). Among them, only CTLA-4 and OX40 (coded by *Tnfrsf4*) were cell surface molecules associated with costimulation, and highly expressed in these mutant and WT Tregs according to the microarray data (*SI Appendix*, Fig. S5 *A* and *B*), suggesting that both molecules might be ones of the responsible candidates expressed in Tregs for the peripheral disappearance.

### The OX40-OX40L, but Not the CTLA4-CD80/86, Interaction Is Required for the Peripheral Disappearance of Dsg3H1-*Rag2*^−/−^ T Cells.

Next, we assessed expression levels of CTLA-4 and OX40 in Tregs before and after the peripheral disappearance. As results, OX40, but not CTLA-4, was up-regulated in Tregs after adoptive transfer of Dsg3H1-*Rag2*^−/−^ T cells into WT mice (Fig. 4 *C* and *D*). This OX40 up-regulation was further assessed in Dsg3-specific Tregs with Dsg3^1-15^:I-A^b^ tetramer. Indeed, the proportion of OX40-expressing Dsg3-specific Tregs increased after adoptive transfer of Dsg3H1-*Rag2*^−/−^ T cells (*SI Appendix*, Fig. S5 *D* and *E*).

Next, we evaluated functional responsibility of OX40 or CTLA-4 in Tregs for the peripheral disappearance in vivo. If Tregs use OX40 or CTLA-4 to suppress autoimmunity and induce T cell disappearance, the two molecules bind the ligands, OX40L or CD80/86, respectively, before Dsg3H1-*Rag2*^−/−^ T cell disappearance. To determine which molecular interaction reproduces the Treg-dependent peripheral disappearance of autoreactive CD4^+^ T cells, we administered CTLA4-Ig or anti-OX40L blocking antibody in Treg-ablated mice, to which the same cell number of Ly5.1^+^ Dsg3H1-*Rag2*^−/−^ T cells and CellTrace-labeled Ly5.2^+^CD4^+^ T cells as internal control were adoptively transferred. The CTLA4-Ig and anti-OX40L antibodies would be expected to detect CD80/CD86 and OX40L respectively, principally in antigen-presenting cells (APCs), and to recapitulate Treg CTLA-4 and OX40 actions, if indeed these molecules were responsible for the disappearance. In the Treg-ablated condition, Dsg3H1-*Rag2*^−/−^ T cells usually overcome peripheral disappearance and sequentially induce interface dermatitis (Fig. 3 *F–H*), OX40L antibody treatment significantly restored the T cell disappearance but CTLA4-Ig treatment did not (Fig. 4 *E* and *F* and *SI Appendix*, Table S1).

These results together indicate that the OX40-OX40L interaction, but not the CTLA4-CD80/86 interaction, is one of the crucial pathways involved in the Treg-dependent peripheral disappearance of Dsg3H1-*Rag2*^−/−^ T cells.

### Treg–Dendritic Cell Interaction Reduces OX40L Expression Levels in Dendritic Cells.

Restoration of peripheral disappearance by anti-OX40L blocking antibody in Treg-ablated mice inspired us to explore how OX40-expressing Tregs indirectly inhibited the OX40-OX40L interaction between APCs and T cells, including autoreactive T cells. In one possibility, interaction between Tregs and dendritic cells (DCs) might down-regulate OX40L expression of DCs.

**Fig. 5.**
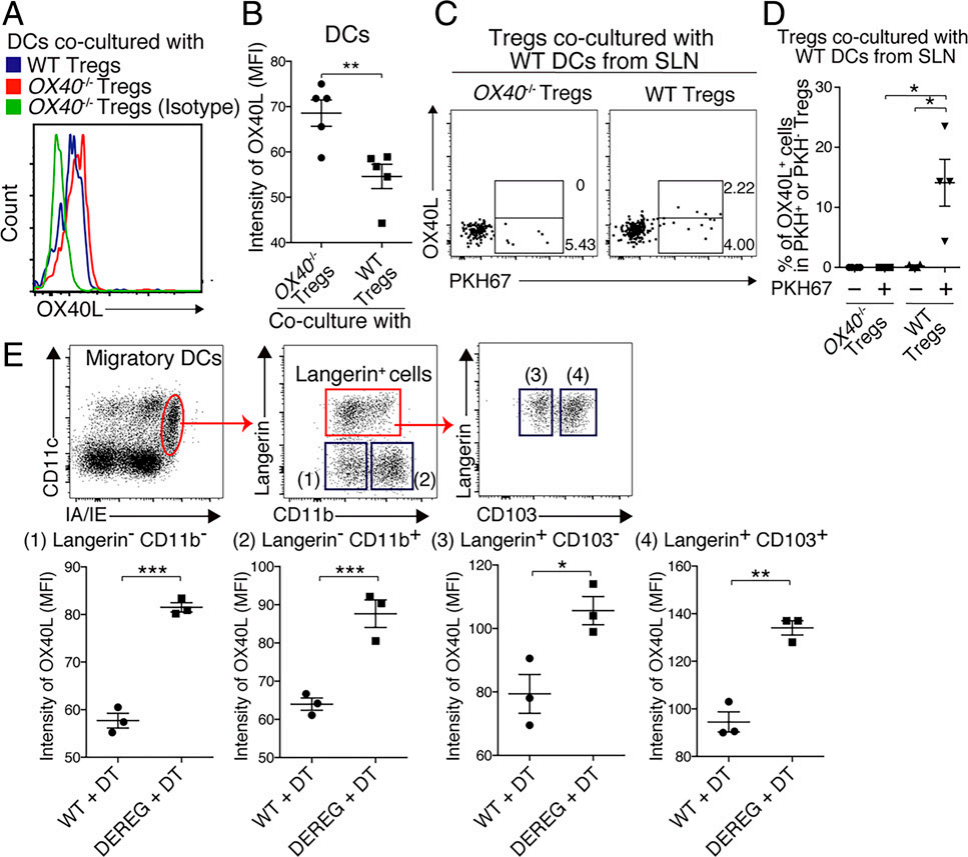
OX40L expression on DCs can be regulated by Tregs. (*A* and *B*) FCM analysis and quantitative summaries of OX40L expression in DCs which were isolated from SLNs of WT mice and cultured with *OX40*^−/−^ or WT Tregs. (*C*) FCM plots of OX40L expression in *OX40*^−/−^ or WT Tregs which were cultured with PKH67-labeled DCs from SLN (gated on CD4^+^CD25^+^Foxp3^+^). Positive fluorescence of PKH67, a lipophilic long chain carbocyanine dye, means that the cells acquired cell membrane from cocultured DCs from SLN of WT mice. OX40L^+^ and OX40L^−^ PKH67^+^ cells are gated by squares; the proportions are shown. (*D*) Quantitative summary of OX40L^+^ cells in PKH67^+^ and PKH67^−^ cells of *OX40*^−/−^ or WT Tregs, which were cultured with PKH67-labeled DCs from SLN of WT mice. (*E*) Representative FCM plots showing four subsets of migDCs in SLN of WT mice (1): Langerin^−^CD11b^−^ (2), Langerin^−^CD11b^+^ (3), Langerin^+^CD103^−^, and (4) Langerin^+^CD103^+^ cells. Quantitative summaries of the OX40L expression levels in each subset in DT-treated WT and DEREG mice are also shown. Means ± SEMs are shown. *P < 0.05, **P < 0.01, and ***P < 0.001, and ****P < 0.0001 based on unpaired *t* tests between groups. Data are from four (*A–D*) or two (*E*) independent experiments (*n* = 3 to 5 per group).

To explore this, we performed in vitro experiments in which SLN-derived DCs were cocultured with WT or *OX40*^−/−^ Tregs. The OX40L expression levels in DCs were slightly but significantly reduced on coculture with WT Tregs, compared to that on coculture with *OX40*^−/−^ Tregs (Fig. 5 *A* and *B*). Furthermore, when DCs were labeled with the cell membrane tracer PKH67 (a lipophilic long-chain carbocyanine dye) before coculture, OX40L expression was detected only in WT Tregs, principally PKH67^+^ WT Tregs, but not *OX40*^−/−^ Tregs (Fig. 5 *C* and *D*). These results indicated that interaction between Tregs and DCs can suppress OX40L expression in DCs. The mechanism of OX40L down-regulation in DCs after coculture with Tregs might involve Treg acquisition of cell membrane components (including OX40L) from DCs in a Treg-derived OX40-dependent manner. Indeed, OX40L was detected on the ex vivo cell surfaces of Tregs from WT but not *OX40*^−/−^ mice (*SI Appendix*, Fig. S5 *F* and *G*). In addition, all subpopulations of CD11c^int^MHCII^high^ migratory DCs, which potentially acquire Dsg3 in the skin and then interact with Dsg3-specific T cells in the SLNs, evidenced constrained expression of OX40L if Tregs were present in vivo during peripheral disappearance (Fig. 5*E*). These in vivo results are consistent with the results of the coculture experiments involving Tregs and DCs.

### Constrained OX40-Signaling Is One of the Crucial Factors to Induce the Peripheral Disappearance of Dsg3H1-*Rag2*^−/−^ T Cells.

**Fig. 6.**
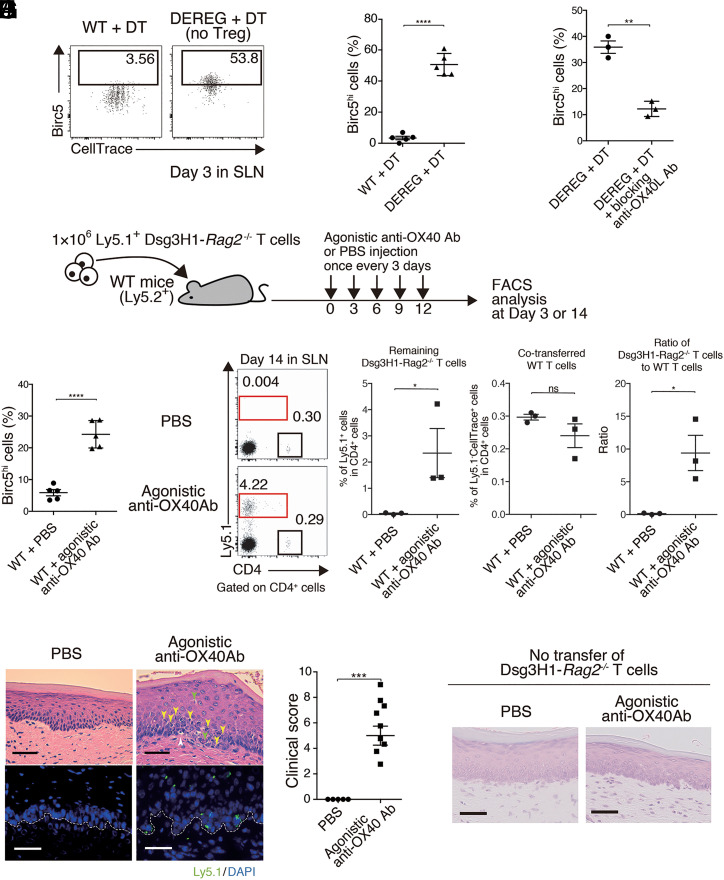
Constrained OX40-signaling in Dsg3H1-*Rag2*^−/−^ CD4^+^ T cells is crucial for their Treg-dependent peripheral disappearance. (*A* and *B*) FCM plots and quantitative summary of Birc5^hi^ Dsg3H1-*Rag2*^−/−^ T cells in SLNs 3 d after transfer to DT-treated WT and DEREG mice (gated on Vβ6^+^Ly5.1^+^CD4^+^). (*C*) Proportion of Birc5^hi^ Dsg3H1-*Rag2*^−/−^ T cells in SLNs 3 d after transfer to WT mice and anti-OX40L Ab-injected WT mice (gated on Vβ6^+^Ly5.1^+^CD4^+^). (*D*) Procedure for the transfer of Dsg3H1-*Rag2*^−/−^ T cells to PBS or agonistic anti-OX40 Ab-injected WT mice. (*E*) Quantitative summary of Birc5^hi^ Dsg3H1-*Rag2*^−/−^ T cells in SLNs 3 d after transfer to PBS or agonistic anti-OX40 Ab-injected WT mice (gated on Vβ6^+^Ly5.1^+^CD4^+^). (*F* and *G*) FCM plots and quantitative summaries of PBS or anti-OX40 Ab-injected WT mice after the transfer of 1 × 10^6^ CellTrace-labeled Ly5.1^+^ Dsg3H1-*Rag2*^−/−^ T cells and 1 × 10^6^ Ly5.2^+^ WT T cells as an internal control (gated on CD4^+^). Proliferated CellTrace^low^Ly5.1^+^Dsg3H1-*Rag2*^−/−^ T cells and CellTrace^+^Ly5.1^−^ cotransferred WT T cells were gated by red and black square. Remaining Dsg3H1-*Rag2*^−/−^ T cell ratio was calculated as the proportion of CellTrace^low^Ly5.1^+^Dsg3H1-*Rag2*^−/−^ T cells divided by that of CellTrace^+^Ly5.1^−^ WT T cells. (*H* and *I*) Pathology of the palate of PBS or anti-OX40 Ab-injected WT mice and clinical score at day 14 after the transfer of Dsg3H1-*Rag2*^−/−^ T cells. For IF, the palate was stained with anti-Ly5.1 Abs (green) and DAPI (blue). Dotted lines indicate the BMZ. In H&E-stained images, infiltrating lymphocytes (yellow arrow), Civatte body (green), and liquefaction (white) are indicated. (Scale bars, 50 μm.) The data of three independent experiments were pooled (*n* = 2 to 4). (*J*) Pathology of the palate of anti-OX40 Ab- or PBS-injected WT mice at day 14 after the initiation of treatment. (Scale bars, 50 μm.) Means ± SEMs are shown. ns, not significant, *P < 0.05, **P < 0.01, ***P < 0.001, and ****P < 0.0001 based on unpaired *t* tests (*B*, *C*, *E*, *G*, and *I*) between the groups. Data are from three (*A–C*, *F*, and *G*) or two (*E* and *J*) independent experiments (*n* = 3 to 5 mice per group)

In the absence of Tregs, unconstrained expression of OX40L in migratory DCs and no peripheral disappearance were observed but OX40L blockade restored them. Since Dsg3H1-*Rag2*^−/−^ T cells interact with DCs in an antigen-specific manner, altered OX40L expression in DCs presumably influences OX40-signaling in proliferating Dsg3H1-*Rag2*^−/−^ T cells after antigen stimulation. To understand the OX40-signaling status, we investigated Birc5, a downstream molecule of OX40 (27), in proliferating Dsg3H1-*Rag2*^−/−^ T cells. Expression of Birc5 in Dsg3H1-*Rag2*^−/−^ T cells was increased under Treg-deficient condition, where Dsg3H1-*Rag2*^−/−^ T cells survive, but hardly in WT mice (Fig. 6 *A* and *B*). Because the up-regulation of Birc5 in Dsg3H1-*Rag2*^−/−^ T cells was suppressed by anti-OX40L blocking Ab injection in Treg-deficient condition (Fig. 6*C*), Birc5 up-regulation depends on a functional OX40-signaling received by Dsg3H1-*Rag2*^−/−^ T cells in vivo. By contrast, anti-OX40 agonistic Ab treatment up-regulated Birc5 expression in proliferating Dsg3H1-*Rag2*^−/−^ T cells after adoptive transfer into WT mice (Fig. 6 *D* and *E*). Strikingly, Dsg3H1-*Rag2*^−/−^ T cells did not disappear, rather inducing interface dermatitis (Fig. 6 *F–I* and *SI Appendix*, Table S1), whereas agonistic Ab treatment alone did not induce dermatitis in WT mice (Fig. 6*J*).

### OX40 Signaling in Dsg3H1-*Rag2*^−/−^ T Cells Is Necessary for Cell Survival.

**Fig. 7.**
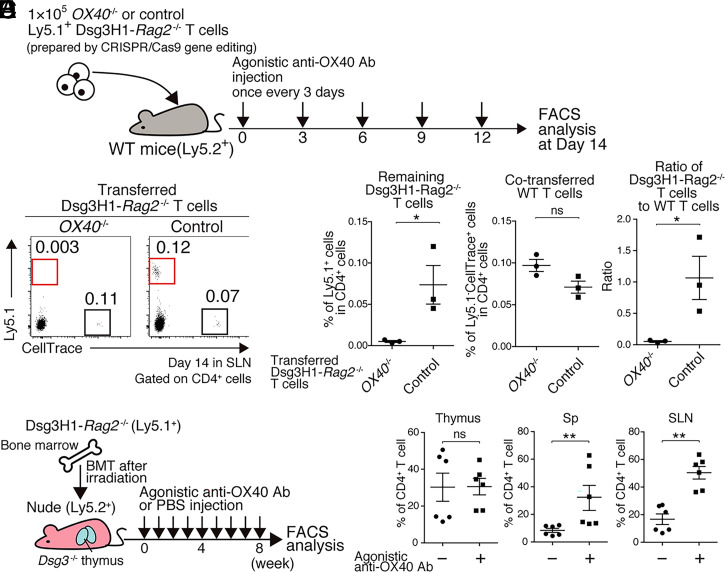
Peripheral deletion was achieved via loss of OX40 signaling in Dsg3H1-*Rag2*^−/−^ T cells. (*A*) Outline of *OX40*^−/−^ Dsg3H1-*Rag2*^−/−^ T cell transfer experiment. *OX40*^−/−^ or control Ly5.1^+^ Dsg3H1-*Rag2*^−/−^ T cells were prepared by CRISPR/Cas9 genome editing with OX40 or negative control guide RNA (gRNA). The cells were transferred into anti-OX40 Ab-treated WT mice. (*B* and *C*) FCM plots of lymphocytes in SLNs of the recipient mice on day 14 after transfer of 1 × 10^5^ cells Ly5.1^+^ *OX40*^−/−^ or control Dsg3H1-*Rag2*^−/−^ T cells and 1 × 10^5^ cells Ly5.2^+^ WT T cells (an internal control), both of which were CellTrace-labeled (gated on CD4^+^). Proliferated CellTrace^low^Ly5.1^+^Dsg3H1-*Rag2*^−/−^ T cells and CellTrace ^+^Ly5.1^−^ cotransferred WT T cells were gated by red and black squares, respectively. Remaining Dsg3H1-*Rag2*^−/−^ T cell ratio was calculated as the proportion of CellTrace^low^ Ly5.1^+^Dsg3H1-*Rag2*^−/−^ T cells divided by that of CellTrace^+^Ly5.1^−^ WT T cells. Data are from two independent experiments (*n* = 3 mice per group). (*D*) Outline of the anti-OX40 Ab injection experiment. Thymus-transplanted chimeric mice underwent BMT from Ly5.1-Dsg3H1-*Rag2*^−/−^ mice, followed by administration of the indicated Abs. (*E*) Proportion of Dsg3H1-*Rag2*^−/−^ T cells in the thymus, SLNs, and Sp of PBS- or anti-OX40 Ab-injected mice (*n* = 6, data pooled from two independent experiments). Means ± SEMs are shown. ns, not significant, *P < 0.05, and **P < 0.01 based on unpaired *t* tests (*C* and *E*) between the groups.

After Treg-ablation or agonistic anti-OX40 Ab injection that allowed Dsg3H1-*Rag2^−/−^* T cells to survive, OX40 signaling of cells other than Dsg3H1-*Rag2^−/−^* T cells might have been affected, and it thus remained unclear whether OX40 signaling in Dsg3H1-*Rag2^−/−^* T cells was important in terms of disappearance. To explore a possibly indispensable role for OX40 signaling in terms of Dsg3H1-*Rag2^−/−^* T cell peripheral disappearance, we knocked-out the *OX40* gene of Dsg3H1-*Rag2^−/−^* T cells using the CRISPR/Cas9 technique. Loss of OX40 expression in CRISPR/Cas9-treated *OX40*^−/−^ T cells, but not control T cells, was confirmed (*SI Appendix*, Fig. S6*A*); the control CD69 expression levels were comparable in both cells (*SI Appendix*, Fig. S6*B*). In WT mice under agonistic anti-OX40 Ab treatment, Dsg3H1-*Rag2^−/−^* T cells usually survived, but they disappeared and interface dermatitis was not induced when *OX40* in the T cells were knocked-out (Fig. 7 *A–C* and *SI Appendix*, Table S1). These results indicated that loss of OX40 signaling in autoreactive T cells triggered the disappearance.

Finally, we showed that enhanced OX40-signaling cancelled the peripheral disappearance of Dsg3H1-*Rag2*^−/−^ T cells even in the *Dsg3*^−/−^ thymus transplantation model (Fig. 7*D*). In fact, Dsg3H1-*Rag2*^−/−^ T cell proportions in the Sp and SLNs, but not the thymus, were significantly increased by an anti-OX40 agonistic Ab (Fig. 7*E*). Therefore, an enhanced OX40-signaling can overcome the peripheral disappearance, a physiological mechanism for avoiding autoimmunity in WT mice.

### OX40 on Tregs Is Crucial in Terms of the Peripheral Disappearance of Dsg3H1-*Rag2*^−/−^ T Cells.

**Fig. 8.**
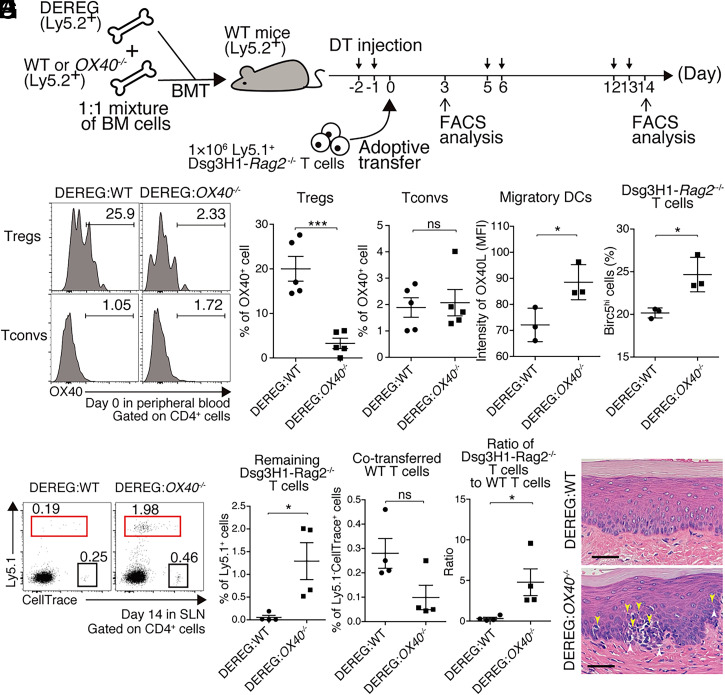
The lack of *OX40* in Tregs disturbs constraints on OX40 signaling and the peripheral disappearance of Dsg3H1-*Rag2*^−/−^ T cells. (*A*) An outline of the experiment featuring generation of mixed-BM chimeric mice receiving DEREG and *OX40^−/−^* or WT BM cells, and evaluation of Dsg3H1-*Rag2*^−/−^ T cell fates in the absence or presence of Treg OX40. (*B* and *C*) Histogram and quantitative summaries of the Tconvs (CD4^+^Foxp3^−^) and Tregs (CD4^+^CD25^+^Foxp3^+^) in peripheral blood cells of the indicated mice utilizing at 2 d after DT injection. DEREG:WT and DEREG:*OX40^−/−^*, respectively, are WT mice transplanted with 1:1 mixtures of DEREG and WT or DEREG and *OX40*^−/−^ BM cells. (*D* and *E*) Quantitative summary of OX40L expression in migratory DCs (D: CD11c^int^MHCII^high^) and Birc5^hi^ Dsg3H1-*Rag2*^−/−^ T cells (E: Vβ6^+^Ly5.1^+^CD4^+^) of SLNs at 3 d after adoptive transfer. (*F* and *G*) FCM plots and quantitative summaries of Dsg3H1-*Rag2*^−/−^ T cell proportions in SLNs at 14 d after adoptive transfer. For adoptive transfer, 1 × 10^6^ Dsg3H1-*Rag2^−/−^* and 1 × 10^6^ WT T cells, both of which were CellTrace-labeled, were transferred. Proliferated CellTrace^low^Ly5.1^+^ Dsg3H1-Rag2^−/−^ T cells and Ly5.2^+^ WT CD4^+^ T cells as an internal control were gated by red and black squares, respectively. Remaining Dsg3H1-*Rag2*^−/−^ T cell ratio was calculated as the proportion of CellTrace^low^Ly5.1^+^Dsg3H1-*Rag2*^−/−^ T cells divided by that of Cell Trace^+^Ly5.1^−^ WT T cells. (*H*) Pathology of the palate at day 14 after adoptive transfer of Dsg3H1-*Rag2*^−/−^ T cells. In H&E-stained images, infiltrating lymphocytes (yellow arrowheads) and liquefaction degeneration (white arrowheads) are indicated. (Scale bars, 50 μm.) Means ± SEMs are shown. ns, not significant, *P < 0.05, and ***P < 0.001 based on unpaired *t* tests between the groups. Data are from two independent experiments (*n* = 3 to 7 mice per group).

Finally, to investigate the contribution of Treg OX40 to the peripheral disappearance of Dsg3H1-*Rag2*^−/−^ T cells, we generated mixed-BM chimeras; lethally irradiated WT mice were reconstituted with 1:1 mixtures of DEREG and *OX40*^−/−^ bone marrow (BM) cells. Control mice were reconstituted with DEREG and WT BM cells. After DT injection into recipients of DEREG and *OX40*^−/−^ BM cells, Tregs derived from DEREG BM cells were supposed to be deleted and Tregs derived from *OX40*^−/−^ BM cells were supposed to remain, whereas the BM-derived immune cell populations, other than Treg, could express OX40 given that they were mixed populations of *OX40^+/+^* and *OX40^−/−^* cells. In contrast, residual Tregs were supposed to express OX40 because they were of *OX40^+/+^* status after DT injection in the recipients of DEREG and WT BM cells (Fig. 8*A*). A comparison of the two conditions allowed evaluation of the contribution of OX40 on Tregs to peripheral disappearance after adoptive transfer of Dsg3H1-*Rag2*^−/−^ T cells to mixed-BM chimeric recipients.

As results, there was no significant difference in OX40 expression on Tconvs between the two conditions, while OX40 expression on Tregs were significantly lower in the recipients of DEREG and *OX40*^−/−^ BM cells after DT injection as expected (Fig. 8 *B* and *C*). When Dsg3H1-*Rag2*^−/−^ T cells were adoptively transferred into these mice, higher OX40L expression of migratory DCs and higher Birc5 expression of transferred T cells were observed on day 3 in recipients of DEREG and *OX40*^−/−^ BM cells compared to recipients of DEREG and WT BM cells (Fig. 8 *D* and *E*). On day 14, transferred Dsg3H1-*Rag2*^−/−^ T cells remained and tissue inflammation was histologically apparent (Fig. 8 *F–H*).

To further determine the effects of Treg OX40 to the peripheral disappearance of Dsg3H1-*Rag2*^−/−^ T cells, *Foxp3^Cre-ERT2^-OX40^flox/flox^* mice were generated (*SI Appendix*, Fig. S7*A*). In these mice, OX40 expression level of Tregs was decreased after tamoxifen (TAM) treatment (*SI Appendix*, Fig. S7 *B* and *C*). After daily treatment of TAM for 5 d, Dsg3H1-*Rag2*^−/−^ T cells were adoptively transferred into *Foxp3^Cre-ERT2^-OX40^flox/flox^* mice; Birc5 expression was up-regulated on day 3 and the cell populations remained rather high, inducing tissue inflammation that was histologically and clinically apparent on day 14 (*SI Appendix*, Fig. S7 *D–G*, *n* = 2). These results were consistent with the findings of the aforementioned mixed-BM chimera mice in terms of the crucial roles of OX40 in Treg on the peripheral disappearance of Dsg3H1-*Rag2*^−/−^ T cells (Fig. 8 *E–H*).

Taken all together, findings presented here clearly demonstrated that peripheral tolerance has a potent function that eliminates autoreactive CD4^+^ T cells in an antigen-specific manner under physiological condition of WT mice even if central tolerance is imperfect. This mechanism is crucially mediated by Tregs, especially OX40 on the Tregs is one of the important factors for the peripheral disappearance, constraining OX40-signaling in autoreactive pathogenic T cells.

## Discussion

Using thymus transplantation and adoptive transfer models, we show the existence of peripheral tolerance to Dsg3, a nonartificial autoantigen in the epidermis, and dissect a part of the cellular and molecular mechanisms that prevent autoreactivity of Dsg3-specific T cell via inducing the deletion to keep the subjects healthy under physiological conditions. The thymus transplantation model provided a unique immunological situation in which tolerance functioned only in the periphery in a Dsg3-specific manner without modifying expression of Dsg3 as the peripheral antigen. Furthermore, the adoptive transfer model not only reconfirmed the existence of Dsg3-specific peripheral tolerance but also elucidated the mechanism at the cellular and molecular levels using a variety of recipients (*SI Appendix*, Table S1).

There are few previous reports that analyzed peripheral immune reaction utilizing thymus transplantation. In the model using melanocyte tyrosinase as an antigen, *tyrosinase^−/−^* thymus was transplanted to thymectomized mice and lack of splenic T cell activity was observed after infection with tyrosinase-bearing recombinant virus (28). Different from the previous study, our experimental system, utilizing thymus transplantation, was able to assess the tolerance mechanism in the periphery under the physiological condition without influence from central tolerance. In addition, we identified the peripheral deletion in a unique way.

Peripheral tolerance identified in this study was mediated by the peripheral disappearance of Dsg3-specific CD4^+^ T cells, which requires Treg function. We showed that even Tregs severely affected by the *Foxp3^R397W^* mutation were sufficient to cause peripheral disappearance of autoimmune Dsg3-specific T cells. To identify one of the crucial pathways that link between responsible Tregs and affected Dsg3H1-*Rag2*^−/−^ T cells, we initially searched for Treg-specific genes that remained expressed in these *Foxp3^R397W^*-mutated Treg cells and found that the OX40 was one of the candidates for regulating peripheral disappearance. Indeed, our in vitro study revealed that OX40 in Tregs is crucial to lower OX40L expression in DCs that are expected to interact with autoreactive T cells in an antigen-specific manner. Moreover, results from experiments blocking or stimulating the signal implied that the OX40-OX40L axis between T cells and DCs is one of the critical pathways for peripheral tolerance to Dsg3. Additionally, the disappearance of *OX40*^−/−^ Dsg3H1-*Rag2*^−/−^ T cells, prepared via CRISPR/Cas9 gene editing, under anti-OX40 agonistic Ab-injected condition, demonstrated that OX40 signaling in Dsg3H1-*Rag2*^−/−^ T cells was specifically required for survival. Furthermore, experiments utilizing mixed-BM chimeric mice and Treg-specific *OX40* conditional knockout (cKO) mice in which almost all Tregs lacked *OX40* implied that the peripheral disappearance was, at minimum, regulated by OX40 on Tregs, thus revealing a function of Tregs.

The roles of OX40 in Tregs are minimally understood (29, 30). Forced expression of Foxp3 in conventional T cells alone does not reproduce Treg-specific epigenetic features (31); most of the Treg-specific superenhancer (Treg-SE) landscape is determined by Satb1 during thymic Treg development irrespective of Foxp3 (32). OX40 is governed by Treg-SE and is down-regulated by Satb1 deficiency in Tregs. Indeed, OX40 expression is reportedly maintained even in severely affected mutant Tregs that result in systemic inflammation indistinguishable from that of *Foxp3*^−/−^ mice (33). Although we have only just begun to understand the epigenetic changes involved in Treg development, these results including our findings together indicate that OX40, whose expression is epigenetically programmed irrespective of Foxp3, may be a crucial Treg molecule in peripheral tolerance.

We observed three things during the peripheral disappearance: OX40 levels were up-regulated in Tregs as well as Dsg3-specific Tregs; OX40L up-regulation in migratory DCs was constrained; and OX40-signaling in Dsg3H1-*Rag2*^−/−^ T cells was suppressed. The latter two observations were not valid in the Treg-deficient condition and mixed-BM chimeric mice in which almost all Tregs lacked *OX40,* and the last was also invalid in Treg-specific *OX40* cKO mice. It may be partly mediated via interaction between Tregs and DCs as shown in our coculture experiments (Fig. 5 *A–D*). These findings indicate that autoreactive CD4^+^ T cells, migratory DCs, and Tregs could be intricately connected to the peripheral disappearance of Dsg3H1-*Rag2*^−/−^ CD4^+^ T cells, adjusting the intensity of the OX40-signaling as a part of the underlying mechanisms.

Regarding the roles of OX40 in immunological peripheral tolerance, only restoration of anergic state in CD4^+^ and CD8^+^ T cells by agonistic OX40-signaling was previously demonstrated (34–36), but roles of OX40 in deletional tolerance had been unknown. Induction of peripheral T cell deletion in the absence of OX40-signaling was identified in our study. Such deletion should be a robust and decisive immune tolerance mechanism because it physically eliminates the risk of reactivation of autoreactive T cells. In this sense, our data highlight an important role for OX40 in tolerance.

Antigen presentation of Dsg3 by migratory DCs is the first step in the peripheral disappearance of proliferating Dsg3-specific T cells. It is puzzling why autoreactive T cells have to proliferate before deletion. This process is partly supported by a previous study that used H^+^/K^+^ ATPase-specific TxA23 CD4^+^ T cells. These T cells potentially induced autoimmune gastritis after transfer to lymphopenic mice but proliferated before deletion in paragastric LN after transfer to WT mice (37). Our results and a previous study indicate that antigen presentation is necessary to not only initiate the tolerogenic process but also give antigen specificity to this process. In addition, the presence or absence of Tregs determines completely different immunological outcomes after T cell proliferation (i.e., constraint vs. induction of autoimmunity), indicating a central role of Tregs in this deletional mechanism.

Possible mechanisms by which Treg OX40 can constrain OX40-signaling in Dsg3H1-*Rag2*^−/−^ T cells are competition- and trogocytosis-based processes. Both processes need Dsg3H1 T cells, Dsg3 antigen-presenting APCs, and Tregs to colocalize. OX40L on APCs may be competitively occupied by up-regulated OX40 on Tregs and subsequently lose the chance to initiate OX40-OX40L signaling into Dsg3H1-*Rag2*^−/−^ T cells after the ligation. In contrast, trogocytosis is known as a process in which T cells extract surface molecules during antigen-specific contact with APCs (38, 39). In addition, Treg acquisition of CD86 from DCs via CTLA-4 has been demonstrated as one of the immunoregulatory function of CTLA-4 (40), and it is no wonder that OX40L on DCs is subject to trogocytosis by Tregs. Our in vitro coculture experiments elucidated that OX40L was predominantly enriched in PKH67^+^ WT Tregs that acquired cell membrane component from DCs, and, therefore, supported the possibility of trogocytosis as the mechanism to down-regulate OX40L in DCs, at least (Fig. 5 *A–D*).

Although our study provides mechanistic insights into the peripheral deletional tolerance of autoreactive CD4^+^ T cells, we must note several limitations. As the Dsg3H1-*Rag2*^−/−^ T cells used in this study express high-avidity TCR (14), the immunological responses of such transgenic T cells may not be identical to those of autoreactive T cells. Ideally, we should have used not only transgenic T cells but also polyclonal autoreactive T cells. However, Dsg3-specific polyclonal T cells could not be detected by the Dsg3^1-15^:I-A^b^ tetramer in the absence of rDsg3 immunization to expand T cells and allow better visualization. In addition, the fate of Dsg3H1-*Rag2*^−/−^ T cells will have been affected by the large numbers thereof in the adoptive transfer model and their relatively high chimeric ratio in the thymus-transplanted chimeric model. It has been reported that when the number of adoptively transferred cells is high (e.g., 3 × 10^5^ cells), the viable cell numbers decrease more rapidly and the fate of transferred cells changes depending on the dosage (41). As the number of cells that we administered was high compared to that of the previous study, we must consider the possibility that the large number of cells affected their fate. These issues need to be overcome in future. Also, the phenomenon that we observed does not necessarily occur in all T cells. However, our analysis using a Dsg3-specific T cell clone allowed us to identify a T cell phenomenon that is an attractive therapeutic target.

The usefulness of Tregs for clinical immune regulation is gaining attention. Adoptive Treg cell therapy has been used in GVHD and allograft rejection (42, 43), and clinical trials targeting autoimmune diseases such as type 1 diabetes mellitus are under way (44). These therapies utilize Treg preparation protocols such as polyclonally expanded tTregs or iTregs. On the other hand, our data reveal that Tregs are important in regulating Dsg3-specific T cells. Since Dsg3-specific CD4^+^ T cells can be detected in PV patients with an MHCII tetramer (45), the recent discovery of a compound that converts effector T cells to Tregs by inhibiting CDK8/19 in mice (46) would make possible the preparation of Dsg3-specific Tregs for antigen-specific Treg therapy of PV.

In conclusion, we have demonstrated the existence of peripheral tolerance to induce Dsg3-specific T cells disappearance, using a thymus transplantation model with divergent expression of autoantigen between the thymus and periphery. In terms of the underlying mechanisms, Treg OX40 played a pivotal role in constraining OX40-signaling in autoreactive T cells prior to their disappearance. Elucidation of this mechanism suggests promising therapeutic approaches targeting Treg or OX40-OX40L signaling for control of T cell-mediated autoimmune diseases.

## Materials and Methods

Detailed experimental methods including those for flow cytometry, transplantation of thymus and BM, adoptive transfer, injection of reagents, coculture experiments, CRISPR/Cas9 gene editing, generation of OX40-floxed mice, and histological analyses appear in *SI Appendix*. The Keio University Ethics Committee and RIKEN ethics committee for Animal Experiments approved all of the experiments in this study.

## Supplementary Material

Supplementary File

## Data Availability

Transcriptome data utilized for Fig. 4*B* are available in the Gene Expression Omnibus database, https://www.ncbi.nlm.nih.gov/geo/ (GSE89744). All other study data are included in the article and/or *SI Appendix*.

## Change History

April 8, 2022: The article license has been updated.
