# Supplementary material for: Peripheral tolerance by Treg via constraining OX40 signal in autoreactive T cells against desmoglein 3, a target antigen in pemphigus
Source: Proc Natl Acad Sci U S A. 2021 Nov 30;118(49):e2026763118. doi: 10.1073/pnas.2026763118 (PMC8670434; doi:10.1073/pnas.2026763118)
Supplement: Supplementary File [file pnas.2026763118.sapp.pdf]

SI Appendix

Peripheral tolerance by Treg via constraining OX40 signal in autoreactive T cells against desmoglein 3, a target antigen in pemphigus

Authors:

Hisato Iriki[a], Hayato Takahashi[a,1], Naoko Wada[a], Hisashi Nomura[a], Miho Mukai[a], Aki Kamata[a], Hiromi Ito[a], Jun Yamagami[a], Takeshi Matsui[b,c], Yutaka Kurebayashi[d], Setsuko Mise-Omata[e], Hiroshi Nishimasu[f,g], Osamu Nureki[f], Akihiko Yoshimura[e], Shohei Hori[h], and Masayuki Amagai[a,b,1]

Affiliations:

[a] Department of Dermatology, Keio University School of Medicine, 35 Shinanomachi, Shinjuku-ku, Tokyo 160-8582, Japan

[b] Laboratory for Skin Homeostasis, RIKEN Center for Integrative Medical Sciences, 1-7-22 Suehiro-cho, Tsurumi-ku, Yokohama City, Kanagawa 230-0045, Japan

[c] Laboratory for Evolutionary Cell Biology of the Skin School of Bioscience and Biotechnology, Tokyo University of Technology, Tokyo 192-0982, Japan

[d] Department of Pathology, Keio University School of Medicine, 35 Shinanomachi, Shinjuku-ku, Tokyo 160-8582, Japan

[e] Department of Microbiology and Immunology, Keio University School of Medicine, 35 Shinanomachi, Shinjuku-ku, Tokyo 160-8582, Japan

[f] Department of Biological Science, Graduate School of Science, The University of Tokyo, 7-3-1 Hongo Bunkyo-ku, Tokyo 113-0033, Japan

[g] Structural Biology Division, Research Center for Advanced Science and Technology, The University of Tokyo, Tokyo 153- 8904, Japan

[h] Graduate School of Pharmaceutical Sciences, The University of Tokyo, 7-3-1 Hongo Bunkyo-ku, Tokyo 113-0033, Japan

[1]**Correspondence should be addressed to**:

Masayuki Amagai, M.D., Ph.D. (amagai@keio.jp) and

Hayato Takahashi, M.D., Ph.D. (hayato_takahashi@keio.jp)

Department of Dermatology, Keio University School of Medicine

35 Shinanomachi Shinjuku-ku Tokyo 160-8582, Japan

TEL: +81-3-5363-3822 FAX: +81-3-3351-6880

Materials and Methods

Mice

C57BL/6J mice, Ly5.1. WT C57BL/6 inbred mice, *MHCII*$^{-/-}$ mice (Jackson Laboratory), C57BL/6 *Rag2*$^{-/-}$ mice (Central Institute for Experimental Animals), B6 nude mice (Taconic), Dsg3H1-tg mice (1), *Dsg3*$^{-/-}$ mice (2), *Aire*$^{-/-}$ mice (3), DEREG mice (4), *Foxp3*$^{I363V:hCD2}$ mice, *Foxp3*$^{A384T:hCD2}$ mice, *Foxp3*$^{R397W:hCD2}$ mice (5), *Tbx21*$^{-/-}$ mice (6), *OX40*$^{-/-}$ mice (7), *Foxp3*$^{Cre\text{-}ERT2}$ mice (Rodent Model Resource Center; RMRC No. 13134), and *OX40*-floxed mice were bred in the experimental animal care facility at Keio University and RIKEN under specific pathogen–free conditions. For all experiments, 4 - 12-week old male and female mice were utilized.

Antibodies and flow cytometry

A single-cell suspension of the thymus, Sp, or LNs of mice and cultured cells were stained with antibodies purchased from Thermo Fisher, BioLegend, and BD Biosciences. For flow cytometry (FCM), fluorescein isothiocyanate-conjugated anti-mouse CD11c (1:200, N418, BioLegend), allophycocyanin-cyanin 7-conjugated anti-mouse CD4 (1:200, RM4-5, BioLegend), Alexa flour® 488-conjugated anti-mouse CD4 (1:100, GK1.5, BioLegend), allophycocyanin-conjugated anti-mouse CD4 (1:200, GK1.5, BioLegend), Brilliant Ultra Violet™ 395-conjugated anti-mouse CD4 (1:200, RM4-5, BD Biosciences), Brilliant Ultra Violet™ 395-conjugated anti-mouse CD11b (1:200, M1/70, BD Biosciences),fluorescein isothiocyanate-conjugated anti-mouse Ly5.2 (1:200, 104, BioLegend), allophycocyanin-conjugated anti-mouse Ly5.2 (1:200, 104, BioLegend), Brilliant Violet™ 510-conjugated anti-mouse Ly5.2 (1:200, 104, BioLegend), phycoerythrin-cyanin 7-conjugated anti-mouse CD8a (1:200, 53-6.7, BioLegend), fluorescein isothiocyanate-conjugated anti-mouse CD8a (1:200, 53-6.7, BioLegend), Brilliant Violet™ 421-conjugated anti-

mouse CD103 (1:200, 2E7", BioLegend), allophycocyanin-cyanin 7-conjugated anti-mouse IA/IE (1:200, M5/114.15.2, BioLegend), fluorescein isothiocyanate-conjugated anti-mouse IA/IE (1:200, M5/114.15.2, BioLegend), Brilliant Violet™ 510-conjugated anti-mouse CD11b (1:200, M1/70, BioLegend), biotin-conjugated anti-mouse CD25 (1:200, 3C7, BioLegend), phycoerythrin-conjugated anti-mouse Vbeta6 (1:200, RR4-7, BD Biosciences), biotin-conjugated anti-mouse Vbeta6 (1:200, RR4-7, BD Biosciences), allophycocyanin-conjugated anti-mouse Ly5.1 (1:200, A20, BioLegend), Brilliant Violet™ 421-conjugated anti-mouse Ly5.1 (1:200, A20, BioLegend), Brilliant Ultra Violet™ 737-conjugated anti-mouse Ly5.1 (1:200, A20, BD Biosciences), Brilliant Ultra Violet™ 737-conjugated anti-mouse CD11c (1:200, N418, BD Biosciences), biotin-conjugated anti-mouse Ly5.1 (1:100, A20, BioLegend), allophycocyanin-conjugated anti-mouse Foxp3 (1:200, FJK-16s, Thermo Fisher), fluorescein isothiocyanate-conjugated anti-mouse Foxp3 (1:200, FJK-16s, Thermo Fisher), phycoerythrin-cyanin 7-conjugated anti-mouse Foxp3 (1:200, FJK-16s, Thermo Fisher), allophycocyanin-conjugated anti-mouse OX40 (1:200, OX-86, BioLegend), phycoerythrin-conjugated anti-mouse OX40 (1:200, OX-86, BioLegend), Brilliant Violet™ 421-conjugated anti-mouse OX40 (1:200, OX-86, BioLegend), phycoerythrin-conjugated anti-mouse CTLA-4 (1:200, UC10-4F10-11, BD Biosciences), phycoerythrin-conjugated anti-mouse OX40L (1:200, RM134L, BioLegend), allophycocyanin-conjugated anti-mouse Langerin (1:200, 4C7, BioLegend), biotin-conjugated anti-mouse Langerin (1:200, 4C7, BioLegend), Anti-mouse Birc5 (1:100, polyclonal, Novus Biologicals), Allophycocyanin-conjugated anti-mouse Rabbit IgG (1:200, polyclonal, Thermo Fisher), and Phycoerythrin-conjugated anti-mouse Rabbit IgG (1:200, polyclonal, Thermo Fisher) were used. We used 7-AAD Viability Staining Solution (BioLegend) or LIVE/DEAD® Fixable Dead Cell Stains (Thermo Fisher) to discriminate dead cells. Cells were stained for 20 min on ice in staining buffer

(phosphate-buffered saline containing 2% FBS and 5 μg/mL Fcγ R III/II blocking Ab). We performed flow cytometry on a Canto II instrument (BD Biosciences) and analyzed the data using FlowJo (Tree Star).

Intercellular staining

Intracellular staining was performed with antibodies and fixation/permeabilization buffers (BD Biosciences) after treatment with phorbol 12-myristate 13 acetate (Sigma), ionomycin calcium salt (Sigma), and Goldiplug (BD Biosciences). For intracellular Foxp3 staining, fixation/permeabilization buffers from eBioscience were used.

Epidermal and Dermal suspensions

Shaved whole trunk skin was harvested, subcutaneous tissue removed using forceps, and the skin floated (with the epidermal side up) on 10 mL of trypsin-EDTA solution [thus 5 mL of 0.25% (w/v) trypsin (Nacalai Tesque) and 5 mL of 0.05% Trypsin–0.53 mM EDTA·4Na (Nacalai Tesque)] at 37°C for less than 30 min in a 10-cm-diameter dish. The epidermis and dermis were manually separated using forceps. The dermis was manually cut into small pieces with scissors, and further incubated in 4 mL RPMI containing 0.03% (w/v) Liberase TL (Research Grade) (Roche Applied Science) and 200 U/mL DNase (Wako Pure Chemical Industries) for 60 min at 37°C with rotation at 200 rpm (Bioshaker, Taitec). Epidermal and dermal cells were then suspended in 5% (v/v) FBS in PBS, washed, and filtered through a cell strainer (BD Falcon).

Thymus transplantation

The thymus of a neonatal donor mouse was surgically removed and cultured on a porous membrane (Whatman) floating on complete RPMI medium containing 1.35 mM 2'-deoxyguanosine monohydrate (Sigma) for 1 week to deplete preexisting thymocytes in the thymus. Next the thymus was cultured in complete RPMI supplemented with 10% FBS, 1% sodium pyruvate (Thermo Fisher), 1% Glutamax (Thermo Fisher), 1% nonessential amino acid solution (Thermo Fisher), 1% penicillin–streptomycin (Thermo Fisher), and 2 mM 2-mercaptoethanol (Thermo Fisher) for 1 day. The *in vitro*-treated thymus was transplanted beneath the renal capsule of the recipient's kidney. Recipient mice were used for experiments 1 month later.

Bone marrow transplantation

CD4, CD8, and B220-depleted BM cells were prepared by MACS cell seperation system with CD4, CD8, and B220 microbeads (Miltenyi) from Dsg3H1-*Rag2*$^{-/-}$ mice and transferred intravenously into irradiated recipient mice. In the experiments of Figure 1, the radiation dose was 5.5 Gy for nude mice and 7.5 Gy for *Dsg3*$^{-/-}$ mice and WT mice. Recipient mice were used for experiments 2 months later. Chimerism was evaluated by measuring the proportions of Ly5.1^{+} cells in CD4^{-} CD8^{-} thymocytes (Figure. S1D and F).

Generation of mixed-BM chimeras

CD4, CD8, and B220-depleted BM cells of DEREG and either *OX40*$^{-/-}$ or WT mice were mixed at the same cell numbers and injected intravenously into 9.5-Gy-irradiated WT mice.

Adoptive transfer

Dsg3H1-*Rag2*$^{-/-}$ T cells were prepared from the Sp, SLNs, and MLNs of *Dsg3*$^{-/-}$ mice that underwent BMT from Dsg3H1-*Rag2*$^{-/-}$ mice by the depletion of B220$^+$ and CD8$^+$ cells followed by positive selection of Vβ6$^+$ cells using magnetic beads (Miltenyi Biotech) according to the manufacturer's instructions. Control CD4$^+$ T cells were prepared from WT mice by positive selection of CD4$^+$ cells using magnetic beads. For transfer to DT-treated DEREG mice, depletion of CD25$^+$ cells was performed with anti-CD25 Ab-biotin and anti-biotin magnetic beads prior to positive selection of CD4$^+$ cells. The isolated cells (1×10^6 or 1×10^5) were labelled by CellTrace CFSE or CellTrace Violet (Thermo Fisher Scientific) and adoptively transferred into WT or other recipient mice as indicated in each experiment. The clinical severity of skin inflammation by Dsg3H1-*Rag2*$^{-/-}$ T cells was assessed based on the scoring system (Table S2) in some experiments.

Staining with Dsg3$^{1\text{-}15}$:I-A^b tetramer

Dsg3 or 2W1S-specific T cells were detected using the Dsg3$^{1\text{-}15}$:I-A^b or 2W1S:I-A^b tetramer (MBL Life Science) according to a previously described protocol with some modifications (8, 9). The cells were incubated with PE-conjugated tetramer (25 nM) at room temperature for 1 h, followed by washing in 15 mL of ice-cold staining buffer. The tetramer-stained cells were then resuspended in 0.4 mL staining buffer, mixed with 0.1 mL of anti-PE antibody-conjugated magnetic microbeads (Miltenyi), and incubated on ice for 20 min. The cells were then enriched using the MACS cell separating system (Miltenyi) and analyzed via flow cytometry after staining with appropriate antibodies. The reliability of the tetramer staining protocol was confirmed by staining of skin-draining LN cells derived from *Dsg3*$^{-/-}$ mice immunized with recombinant Dsg3 protein (Figure

S5E). The reliability of the tetramer staining protocol was confirmed by staining skin-draining LN cells derived from *Dsg3*$^{-/-}$ mice immunized with recombinant Dsg3 protein (Figure S5C).

Injection of FTY720

FTY720 (30 µg; Cayman Chemical) was administered to WT mice intraperitoneally daily from 24 h before the adoptive transfer of Dsg3H1-*Rag2*$^{-/-}$ T cells until analysis 3 days after adoptive transfer.

Injection of diphtheria toxin

DEREG mice and mixed-BM chimeric mice, thus with BM from DEREG mice, were administered 1 µg diphtheria toxin (Calbiochem) intraperitoneally for two consecutive days per week. The mice were analyzed 2 weeks after the start of administration.

Injection of antibodies

Anti-OX-40L Ab (300 µg; clone RM134L; Bioxcell) diluted in PBS was administered to DT-treated DEREG mice intraperitoneally every 2 days from the day before the adoptive transfer of Dsg3H1-*Rag2*$^{-/-}$ T cells until analyses 3 or 14 days after adoptive transfer. Moreover, 250 µg anti-OX-40 Ab (clone: OX-86; Bioxcell) diluted in PBS was administered to WT mice intraperitoneally every 3 days from the day of the adoptive transfer of Dsg3H1-*Rag2*$^{-/-}$ T cells until analyses 3 or 14 days after adoptive transfer. Anti-PD-1 Ab (250 µg; clone RMP1–14) (10) was administered to WT mice intraperitoneally twice weekly from 1 day before the adoptive transfer of Dsg3H1-*Rag2*$^{-/-}$ T cells until analyses 14 days after adoptive transfer.

Injection of Abatacept

Abatacept (250 µg; E.R. Squibb & Sons) diluted in PBS was administered to DT-treated DEREG mice intraperitoneally every 2 days from the day before the adoptive transfer of Dsg3H1-*Rag2*$^{-/-}$ T cells until analyses 14 days after adoptive transfer.

Coculture experiment

WT and *OX40*$^{-/-}$ Tregs were prepared from SLN of WT and *OX40*$^{-/-}$ mice respectively by positive selection of CD25^{+} cells using magnetic beads (using magnetic beads after employing the CD4^{+} T cell isolation kit). CD11c^{+} DCs were prepared from SLN of WT mice by positive selection of CD11c^{+} cells using magnetic beads. After the positive selection, cell membrane of CD11c^{+} cells were stained with PKH67GL (Sigma-Aldrich). 1×10^4 Tregs and 1×10^4 DCs were co-cultured in 96-well round bottom microwell plates. Twelve hours later, cells were stained with antibodies and analyzed by flow cytometry.

***OX40* knockout in primary T cells**

The recombinant Streptococcus pyogenes Cas9 (SpCas9) protein bearing a nuclear localization was prepared as described previously (11). To prepare SpCas9/guide RNA ribonucleoprotein (RNP) complexes, 450 pmol of crRNA and 450 pmol of trans-activating CRISPR RNA (tracrRNA) were hybridized for 5 minutes at 95°C. The hybridized crRNA/ tracrRNA complex and 180 pmol of purified recombinant SpCas9 protein were preincubated using a P4 Primary Cell 4D-Nucleofector™ X Kit S (Lonza) for 15 minutes at room temperature. Mixture of three OX40 guide RNAs (5’-ACGACAGCACTTGTGACCAC-3’, 5’-GACTGCGTCCAAGCTGTCAC-3’, and 5’-ATCTCCAAGCCTTCCGGAGC-3’) were utilized. Dsg3H1-*Rag2*$^{-/-}$ T cells were

activated by anti-CD3/28 antibody starting on day 0. Forty-eight hours after activation, Dsg3H1-*Rag2*$^{-/-}$ T cells were resuspended in P4 buffer (Lonza). The cell suspensions were then mixed with P4 buffer containing Cas9 RNPs, to a final volume of 20 μL. The 20 μL of cell suspension was electroporated by a 4D-Nucleofector X unit (program code: CM137, Lonza). After electroporation, the cells were transferred into recipient mice after diluted in PBS or cultured with 100 U/mL of human rIL-2 for twenty-four hours.

Generation of *OX40*-floxed-*Foxp3*$^{Cre\text{-}ERT2}$ mice

We developed a conditional knock-out mouse line; we removed *OX40* in a Cre-dependent manner. We purchased a vector targeting *Tnfrsf4* (in which loxP sites flanked the common exons 3 and 4) from the International Mouse Phenotyping Consortium (IKMC project 82469) (Fig. S7A). The targeting vector was electroporated into C57BL/6J x C57BL/6N hybrid ES cells that were subsequently microinjected to form eight-cell-stage embryos, followed by transplantation into the uteri of pseudo-pregnant mice. To remove the neomycin-resistance cassette, newborn mice were crossed with CAG-FLPe mice (12). As this mouse line had lost the loxp sequence at the 3′ side of the targeting site, the loxp sequence of the 3′ side was inserted (using the CRISPR/Cas9 gene editing method) of fertilized eggs of *OX40*$^{incomplete\ flox/incomplete\ flox}$-*Foxp3*$^{Cre\text{-}ERT2}$ mice to generate regular *OX40*$^{flox/flox}$-*Foxp3*$^{Cre\text{-}ERT2}$ mice in which Cre-mediated deletion of exons 3 and 4 created a frameshift mutation in *Foxp3*$^{+}$ Tregs. On PCR genotyping of *OX40*$^{incomplete\ flox/incomplete\ flox}$ mice, the following primers were used to analyze the loxp sequence at the 5′ side: 5′ -CACATGTTGACCATGTGGCCTT-3′ and 5′ -CTGGTCTACATCTGCAGACAG-3′. This detected the WT allele (a 421-bp amplicon). The primers 5′ -TAGCCACAACAGTGGGTGCC-3′

and 5′ -CCGCCTACTGCGACTATAGAG-3′ detected the incompletely floxed allele (a 353-bp amplicon).

Insertion of the loxp sequence of the 3' side into *OX40* gene

100 μM of crRNA (5'-GACTGCGTCCAAGCTGTCAC-3') and 100 μM of tracrRNA were hybridized in 10 μL of Tris-EDTA buffer (10 mM Tris, 0.1 mM EDTA, pH 8.0; Integrated DNA Technologies) for 5 minutes at 95°C, and then placed for 10 minutes at room temperature. In terms of injection method, the hybridized crRNA/ tracrRNA complex (crRNA 40 ng/μl, tracrRNA 60 ng/μl) were incubated with recombinant Cas9 protein (50 ng/μl; Integrated DNA Technologies) and single-stranded oligodeoxynucleotide (ssODN; 6 ng/μl) in Tris-EDTA buffer for 10 minutes at room temperature, followed by injection into the cytoplasm of fertilized eggs of $OX40^{incomplete\ flox/incomplete\ flox}$-$Foxp3^{Cre\text{-}ERT2}$ mice utilizing Cell Tram (Eppendorf) and DMIRB (Leica). In terms of electroporation method, the fertilized eggs of $OX40^{incomplete\ flox/incomplete\ flox}$-$Foxp3^{Cre\text{-}ERT2}$ mice were incubated with the hybridized crRNA/ tracrRNA complex (crRNA 16 ng/μl, tracrRNA 24 ng/μl), recombinant Cas9 protein (100 ng/μl), and ssODN (400 ng/μl) in PBS for 10 minutes at room temperature, and 30 times diluted with Opti-MEM I (Life Technologies), followed by electroporation utilizing Gene Editor GEB15 (BEX). Ultramer DNA Oligos (5'-TGCTATCTAGATGACTGTGATGGACTGGATAAGAAAGCCCTGTGCTTAGGGCATCTG ACATCTGACATCACGAACACCAAGAATTCATAACTTCGTATAGCATACATTATACGA AGTTATCACAGGCTCTACTGTGCAAGGGGCCCCTGCAGATTGAGAGGGGCATGTGA GATCCAAGCCCAGGCCCAGGGACATGTTTG-3'; Integrated DNA Technologies) were

utilized as ssODN. After the injection or the electroporation, the eggs were cultured at 37 ºC under 5% CO_2 in air until 2-cell stage and were implanted into the oviduct of the pseudo-pregnant mice.

Tamoxifen-induced loss of OX40 expression in Tregs

Foxp3$^{Cre\text{-}ERT2}$-*OX40*$^{flox/flox}$ mice generated by re-insertion of the 2nd loxp sequence with CRISPR/Cas9 was identified by detecting loss of OX40 expression in Tregs after tamoxifen injection by flow cytometry (Fig. S7C). Since *Foxp3*$^{Cre\text{-}ERT2}$ mice express Cre-ERT2 fusion protein specifically in Foxp3^{+} Treg. Therefore, tamoxifen treatment activates Cre-dependent deletion of *OX40* gene.

Tamoxifen injection

Tamoxifen (100 µL) (Sigma-Aldrich) diluted in rapeseed oil (20 mg/mL) was intraperitoneally administered to *Foxp3*$^{Cre\text{-}ERT2}$-*OX40*$^{flox/flox}$ mice and littermate mice every day for the first 5 days, followed by adoptive transfer of Dsg3H1-*Rag2*$^{-/-}$ T cells on day 6. Then, tamoxifen was injected every 2 days until analyses at 14 days after adoptive transfer.

Histological analyses

Formalin-fixed paraffin-embedded tissue was cut into serial sections (4 µm thick) and stained with hematoxylin and eosin (H&E). The H&E-stained sections were observed under a BX41 microscope (Olympus). For immunofluorescence staining, 5 µm cryosections of the palate were fixed in 4% paraformaldehyde (Wako) and reacted with anti-mouse Ly5.1 Ab-biotin, anti-CD4 Ab-Alexa Fluor 488, streptavidin-Alexa Fluor 568, and 4,'6-diamidino-2-phenylindole (DAPI;

Sigma). The sections were observed under an Axio Observer Z1 inverted fluorescence microscope (Carl Zeiss).

Supplementary table

Table S1 Summary of Dsg3-specific T cell behavior observed in this study

Mice	Condition	Proliferation	Disappearance	Pathogenicity
WT	Physiplogical	+	+	–
Dsg3$^{-/-}$	No target antigen	–	–	–
MHCII$^{-/-}$	No MHCII-restricted antigen presentation	–	–	–
DT-treated DEREG	No Tregs	+	–	+
DT-treated DEREG with blocking anti-OX40L Ab injection	No Treg & OX40-OX40L signaling blockade	+	+	–
DT-treated DEREG with *OX40*$^{-/-}$ Dsg3H1-*Rag2*$^{-/-}$ T cell transfer	No Treg & no OX40 signaling in Dsg3H1-*Rag2*$^{-/-}$ T cell	NA	+	–
WT with agonistic anti-OX40 Ab injection	Forced enhancement of OX40 signaling	+	–	+
DT-treated DEREG:*OX40*$^{-/-}$	Tregs lack *OX40*	+	–	+ (Epithelial damage was observed.)
TAM-treated *Foxp3*$^{Cre\text{-}ERT2}$-*OX40*$^{flox/flox}$	Tregs lack *OX40*	+	–	+

NA; not assessed.
DEREG:*OX40*$^{-/-}$; WT mice transplanted with a 1:1 mixture of DEREG and *OX40*$^{-/-}$ BM cells.

Table S2 Clinical score

Score	Ears	Tail	Ventral neck and trunk	Back	Face	Limbs
1	Erythema	Scale	Erythema (moderate)	Erythema (moderate)	Erythema	Erythema (moderate)
2	Scale	Crust / erosion (proximal)	Erythema (severe)	Erythema (severe)	Scale	Erythema (severe)
3	Crust / erosion	Crust / erosion (whole)	Scale	Scale	Crust / erosion	Scale
4	Rim defect	Hair loss	Crust / erosion	Crust / erosion	Hair loss	Crust / erosion
5			Hair loss	Hair loss		Hair loss

Supplementary Figure legends

Fig. S1.

A, Outline for generating thymus-transplanted chimeric mice and control mice. Pale red and blue indicate the presence and absence of Dsg3, respectively.

B, FCM plots of peripheral blood cells in nude mice before and 3 weeks after thymus transplantation from WT mice (gated on $CD3^{+}$ cells).

C, Procedure for BMT from Ly5.1-Dsg3H1-*Rag2*$^{-/-}$ mice to thymus-transplanted chimeric mice and control mice.

D, Quantitative summaries of the proportions of $Ly5.1^{+}$ cells among $CD4^{-}$ $CD8^{-}$ cells of the thymi of the mice described in Figure 1D and E (n = 6 or 8; the data of three independent experiments were pooled).

E, FCM plots of recipient-derived thymocytes in WT thymus-transplanted chimeric nude mice 2 months after BMT (gated on $Ly5.2^{+}$ cells). Data are from three independent experiments (n = 3 mice per group).

F, Quantitative summaries of the proportions of $Ly5.1^{+}$ cells among $CD4^{-}$ $CD8^{-}$ cells in the thymi of the mice described in Figure 1A and B. The data are those of two independent experiments (n = 3 mice per group).

Means ± SEMs are shown. ns, not significant based on unpaired t tests between groups.

Fig. S2.

A, FCM plots of lymphocytes in the MLNs, Sp, dermis, and epidermis of WT mice at day 14 after transfer (gated on $V\beta6^{+}$ for MLNs and the Sp, and on $TCR\gamma\delta^{-}$ IA/IE^{-} $V\beta6^{+}$ for the dermis and epidermis). There was no Dsg3H1-*Rag2*$^{-/-}$ T cells, which is gated by square.

B, FCM plots of the SLNs in WT mice after the transfer of $CD4^+$ cells from $Ly5.1^+$WT mice (gated on $V\beta6^+$ cells).

Data are from three independent experiments (n = 2 or 3 mice per group).

Fig. S3.

A, FCM plots of the SLNs of *Aire*$^{-/-}$ and WT mice at day 14 after transfer (gated on $V\beta6^+$ cells). $Ly5.1^+CD4^+$ cells gated by squares are Dsg3H1-*Rag2*$^{-/-}$ T cells (n = 3 mice per group).

B, FCM plots of the SLNs of anti-PD-1 Ab- or PBS-injected WT mice at day 14 after transfer (gated on $V\beta6^+$ cells). $Ly5.1^+CD4^+$ cells gated by squares are Dsg3H1-*Rag2*$^{-/}$ T cells (n = 3 mice per group).

Fig. S4.

A, B, Clinical phenotype (A) and pathology of the palate (B) of DEREG mice two weeks after DT injection. Slight erythema on the nose was clinically observed and mild lymphocytes infiltration is pathologically observed in the submucosa of the palate. Scale bar indicates 100 μm.

C, Clinical phenotype of *Tbx21*$^{-/-}$ mice and *Tbx21*$^{-/-}$*Foxp3*$^{R397W/Y}$ mice at 5 week age without transfer of Dsg3H1-*Rag2*$^{-/-}$ T cells. *Tbx21*$^{-/-}$ mice did not show any skin inflammation and *Foxp*$^{3R397W/Y}$*Tbx21*$^{-/-}$ mice showed severe skin inflammation.

Fig. S5.

A, B, Microarray data of *Ctla4* (A) and *Tnfrsf4* (B) expression. The result was obtained by re-analyzing the data from global gene expression database (GEO; GSE89744).

C, FCM plots of $Dsg3^{1\text{-}15}$:I-A^b and 2W1S:I-A^b tetramer-binding cells in SLNs of recombinant Dsg3-immunized *Dsg3*$^{-/-}$ mice (gated on $CD11b^-B220^-Gr\text{-}1^-CD3^+CD4^+$ cells).

D, E, FCM plots and quantitative summaries of the levels of $Dsg3^{1\text{-}15}$:I-A^b tetramer-binding cells in $OX40^+$ and $OX40^-$ Tregs ($Ly5.1^-CD4^+CD25^+Foxp3^+$) of SLNs before and at 3 days after adoptive transfer of Dsg3H1-*Rag2*$^{-/-}$ T cells.

F, G, FCM plots and quantitative summaries of OX40L expression levels in Tregs of the SLNs of WT and *OX40*$^{-/-}$ mice ($CD4^+CD25^+Foxp3^+$).

Means ± SEMs are shown. *p < 0.05 and **p < 0.01 based on unpaired t-tests between the groups. The data are those of two independent experiments (n = 2 or 3 per group).

Fig. S6.

A, B, FCM plots of the WT T cells to detect OX40 (A) and CD69 (B) expression twenty-four hours after electroporation with SpCas9/guide RNA (gRNA) RNP complexes which contain OX40 or control gRNA.

Fig. S7

A, A schematic of the targeting strategy, the CRISPR/Cas9 gene editing strategy, and the affected allele.

B, An outline of adoptive transfer of Dsg3H1-*Rag2*$^{-/-}$ T cells into Treg-specific *OX40* knock-out mice.

C, A histogram of OX40 expression level on Treg ($CD4^+CD25^+Foxp3^+$) and Tconv ($CD4^+Foxp3^-$) in SLNs at 19 days after initiation of TAM injections and 14 days after adoptive transfer.

D, A histogram of Birc5 expression levels in Dsg3H1-*Rag2*$^{-/-}$ T cells of peripheral blood at 3 days after adoptive transfer.

E, FCM plots of Dsg3H1-*Rag2*$^{-/-}$ T cell proportions in SLNs at 14 days after adoptive transfer. Dsg3H1-*Rag2*$^{-/-}$ T cells and CellTrace-labelled WT CD4^{+} T cells as an internal control were gated by red and black squares, respectively.

F, G, Clinical phenotype and pathology of the palate 14 days after adoptive transfer of Dsg3H1-*Rag2*$^{-/-}$ T cells. In the H&E-stained images, infiltrating lymphocytes (yellow arrow), Civatte body (green), Civatte body (green), and liquefaction (white) are indicated. Scale bars indicate 50 μm. Data are from one experiment (n = 2 per group).

References

1. H. Takahashi *et al.*, Desmoglein 3-specific CD4+ T cells induce pemphigus vulgaris and interface dermatitis in mice. *J Clin Invest* **121**, 3677-3688 (2011).
2. T. Hata *et al.*, Transgenic rescue of desmoglein 3 null mice with desmoglein 1 to develop a syngeneic mouse model for pemphigus vulgaris. *J Dermatol Sci* **63**, 33-39 (2011).
3. N. Kuroda *et al.*, Development of autoimmunity against transcriptionally unrepressed target antigen in the thymus of Aire-deficient mice. *J Immunol* **174**, 1862-1870 (2005).
4. K. Lahl *et al.*, Selective depletion of Foxp3+ regulatory T cells induces a scurfy-like disease. *J Exp Med* **204**, 57-63 (2007).
5. N. Hayatsu *et al.*, Analyses of a Mutant Foxp3 Allele Reveal BATF as a Critical Transcription Factor in the Differentiation and Accumulation of Tissue Regulatory T Cells. *Immunity* **47**, 268-283 e269 (2017).

6. S. Finotto *et al.*, Development of spontaneous airway changes consistent with human asthma in mice lacking T-bet. *Science* **295**, 336-338 (2002).

7. S. D. Pippig *et al.*, Robust B cell immunity but impaired T cell proliferation in the absence of CD134 (OX40). *J Immunol* **163**, 6520-6529 (1999).

8. D. Malhotra *et al.*, Tolerance is established in polyclonal CD4(+) T cells by distinct mechanisms, according to self-peptide expression patterns. *Nat Immunol* **17**, 187-195 (2016).

9. J. J. Moon *et al.*, Naive CD4(+) T cell frequency varies for different epitopes and predicts repertoire diversity and response magnitude. *Immunity* **27**, 203-213 (2007).

10. T. Kanai *et al.*, Blockade of B7-H1 suppresses the development of chronic intestinal inflammation. *J Immunol* **171**, 4156-4163 (2003).

11. H. Nishimasu *et al.*, Crystal structure of Cas9 in complex with guide RNA and target DNA. *Cell* **156**, 935-949 (2014).

12. H. Kanki, H. Suzuki, S. Itohara, High-efficiency CAG-FLPe deleter mice in C57BL/6J background. *Exp Anim* **55**, 137-141 (2006).

Fig. S1.

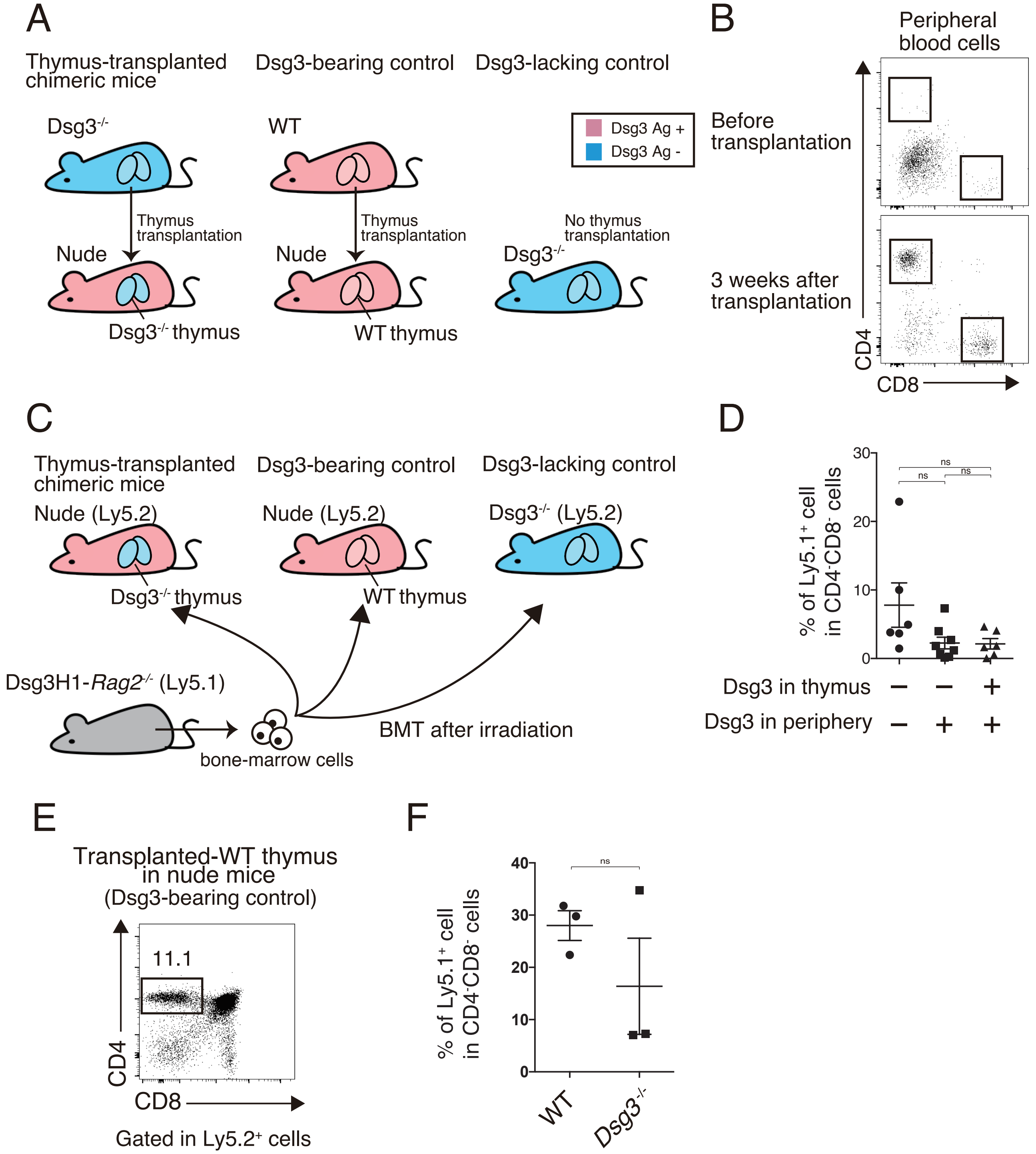
A
Thymus-transplanted chimeric mice
Dsg3-bearing control
Dsg3-lacking control
Dsg3-/-
WT
Dsg3 Ag +
Dsg3 Ag -
Thymus transplantation
No thymus transplantation
Nude
Dsg3-/-
Dsg3-/- thymus
WT thymus
B
Peripheral blood cells
Before transplantation
3 weeks after transplantation
CD4
CD8
C
Thymus-transplanted chimeric mice
Dsg3-bearing control
Dsg3-lacking control
Nude (Ly5.2)
Dsg3-/- (Ly5.2)
Dsg3-/- thymus
WT thymus
Dsg3H1-Rag2-/- (Ly5.1)
bone-marrow cells
BMT after irradiation
D
% of Ly5.1+ cell in CD4-CD8- cells
ns
Dsg3 in thymus
Dsg3 in periphery
E
Transplanted-WT thymus in nude mice (Dsg3-bearing control)
11.1
CD4
CD8
Gated in Ly5.2+ cells
F
% of Ly5.1+ cell in CD4-CD8- cells
ns
WT
Dsg3-/-

Fig. S2.

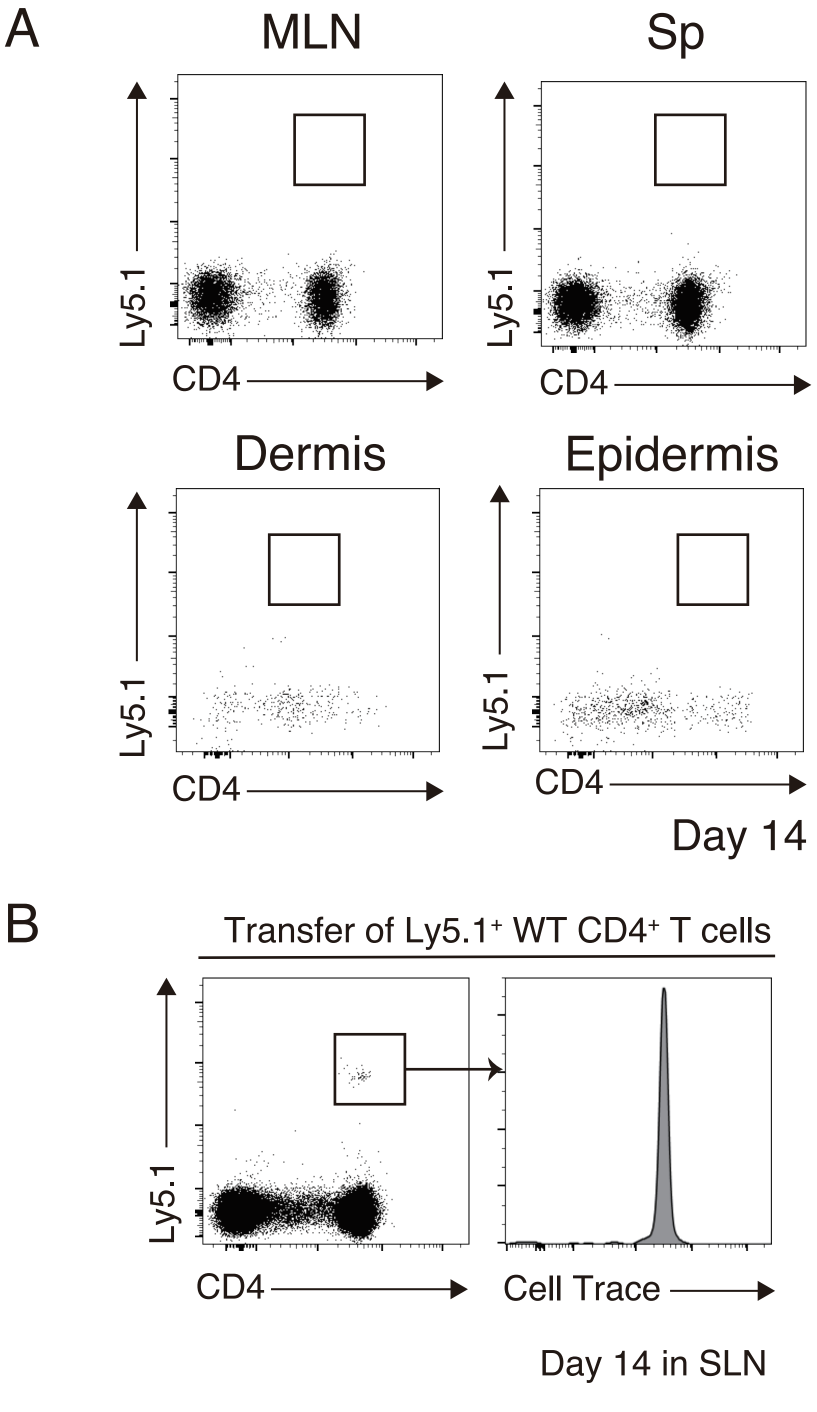
A
MLN
Sp
Dermis
Epidermis
Ly5.1
CD4
Day 14
B
Transfer of Ly5.1+ WT CD4+ T cells
Ly5.1
CD4
Cell Trace
Day 14 in SLN

Fig. S3.

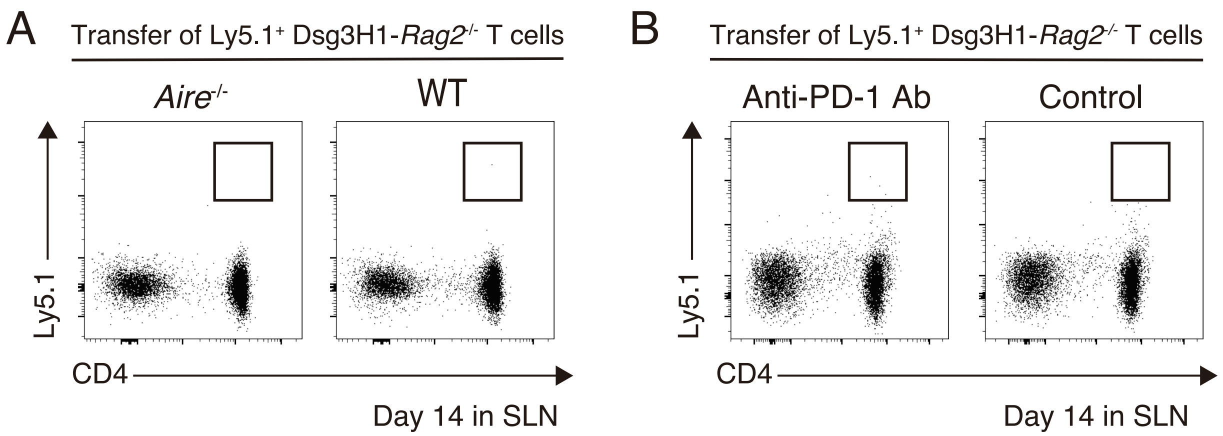

A
Transfer of Ly5.1+ Dsg3H1-Rag2-/- T cells
Aire-/-
WT
Ly5.1
CD4
Day 14 in SLN
B
Transfer of Ly5.1+ Dsg3H1-Rag2-/- T cells
Anti-PD-1 Ab
Control
Ly5.1
CD4
Day 14 in SLN

Fig. S4.

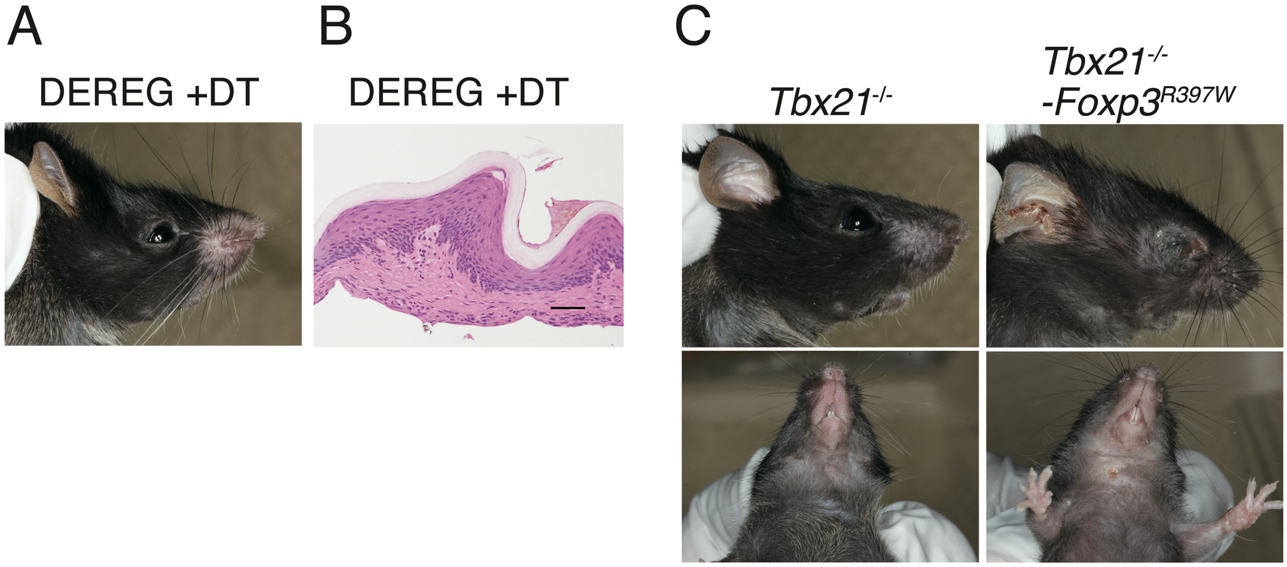

Fig. S5.

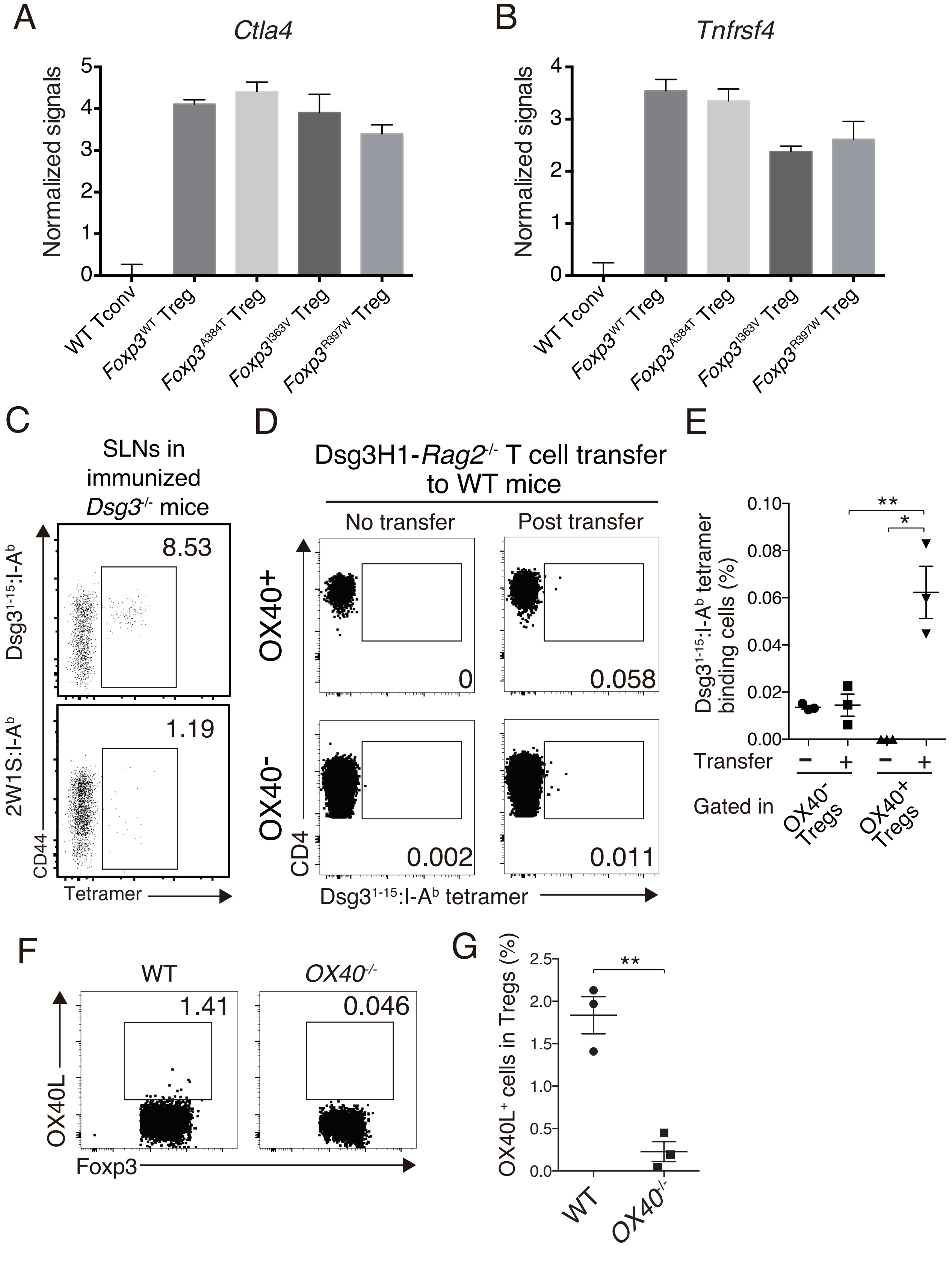
A
Ctla4
Normalized signals
WT Tconv
Foxp3WT Treg
Foxp3A384T Treg
Foxp3I363V Treg
Foxp3R397W Treg
B
Tnfrsf4
C
SLNs in immunized Dsg3-/- mice
Dsg31-15:I-Ab
2W1S:I-Ab
8.53
1.19
CD44
Tetramer
D
Dsg3H1-Rag2-/- T cell transfer to WT mice
No transfer
Post transfer
OX40+
OX40-
0
0.058
0.002
0.011
CD4
Dsg31-15:I-Ab tetramer
E
Dsg31-15:I-Ab tetramer binding cells (%)
Transfer
Gated in
OX40- Tregs
OX40+ Tregs
F
WT
OX40-/-
1.41
0.046
OX40L
Foxp3
G
OX40L+ cells in Tregs (%)
WT
OX40-/-

Fig. S6.

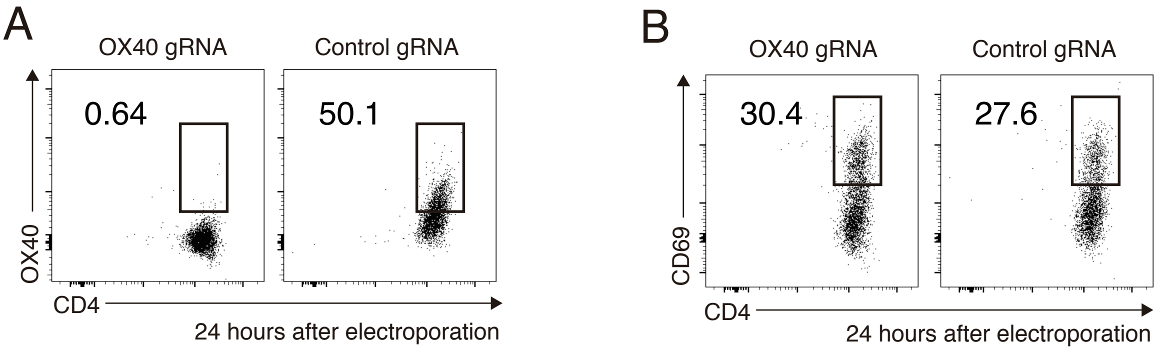

A
OX40 gRNA
Control gRNA
0.64
50.1
OX40
CD4
24 hours after electroporation
B
OX40 gRNA
Control gRNA
30.4
27.6
CD69
CD4
24 hours after electroporation

Fig. S7.

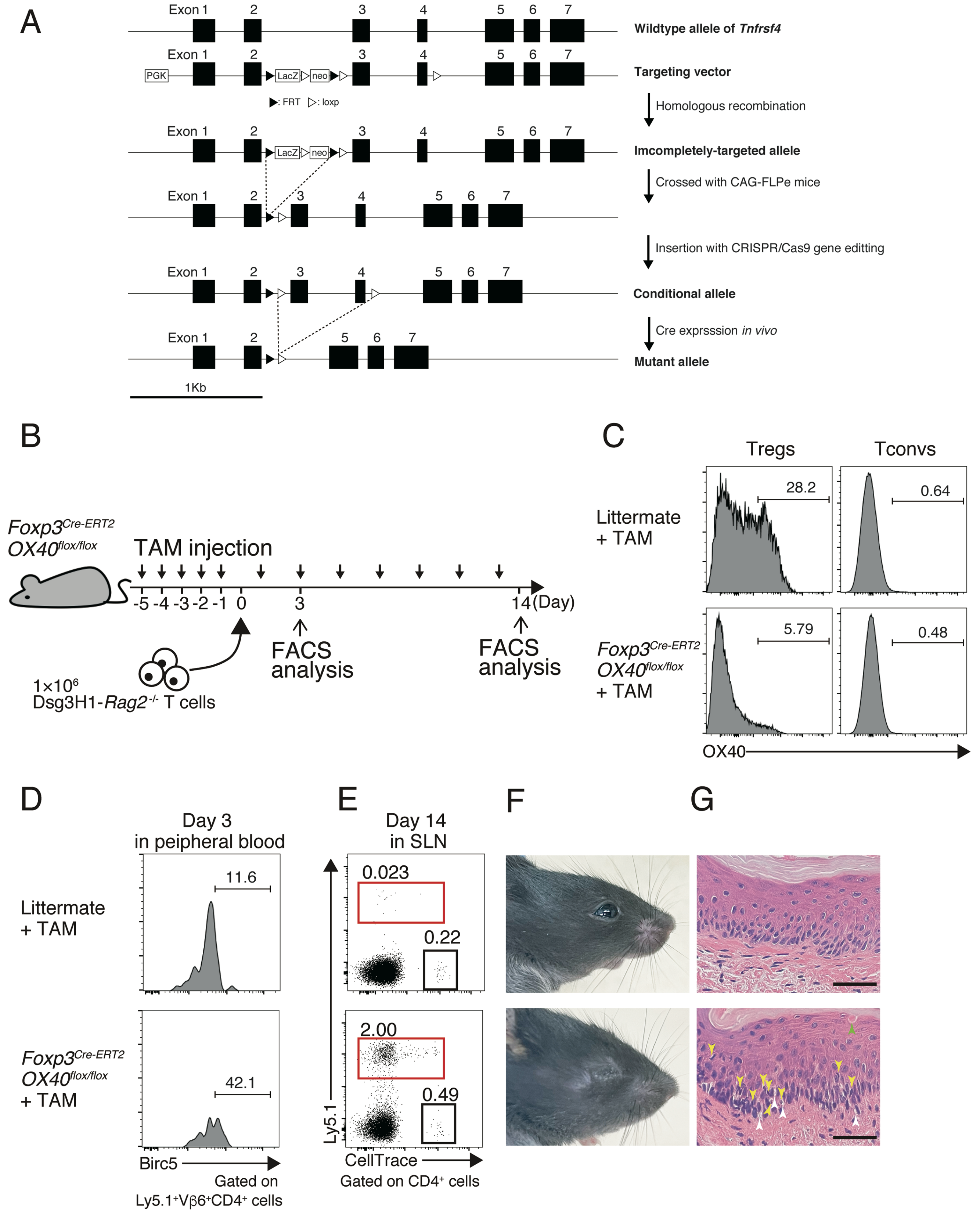
A
Exon 1
2
3
4
5
6
7
Wildtype allele of *Tnfrsf4*
PGK
LacZ
neo
Targeting vector
▶: FRT ▷: loxp
Homologous recombination
Imcompletely-targeted allele
Crossed with CAG-FLPe mice
Insertion with CRISPR/Cas9 gene editting
Conditional allele
Cre exprsssion *in vivo*
Mutant allele
1Kb
B
*Foxp3*Cre-ERT2
*OX40*flox/flox
TAM injection
-5 -4 -3 -2 -1 0 3 14 (Day)
FACS analysis
FACS analysis
1×10⁶
Dsg3H1-*Rag2*-/- T cells
C
Tregs
Tconvs
Littermate + TAM
28.2
0.64
*Foxp3*Cre-ERT2 *OX40*flox/flox + TAM
5.79
0.48
OX40
D
Day 3 in peipheral blood
Littermate + TAM
11.6
*Foxp3*Cre-ERT2 *OX40*flox/flox + TAM
42.1
Birc5
Gated on Ly5.1+Vβ6+CD4+ cells
E
Day 14 in SLN
0.023
0.22
2.00
0.49
Ly5.1
CellTrace
Gated on CD4+ cells
F
G
